# Triage Modeling for Differential Diagnosis Between COVID-19 and Human Influenza A Pneumonia: Classification and Regression Tree Analysis

**DOI:** 10.3389/fmed.2021.673253

**Published:** 2021-08-10

**Authors:** Anling Xiao, Huijuan Zhao, Jianbing Xia, Ling Zhang, Chao Zhang, Zhuoying Ruan, Nan Mei, Xun Li, Wuren Ma, Zhuozhu Wang, Yi He, Jimmy Lee, Weiming Zhu, Dajun Tian, Kunkun Zhang, Weiwei Zheng, Bo Yin

**Affiliations:** ^1^Department of Radiology, Fu Yang No.2 People's Hospital, Fuyang, China; ^2^Key Laboratory of Public Health Safety, Ministry of Education, Department of Environmental Health, School of Public Health, Fudan University, Shanghai, China; ^3^Key Laboratory of Health Technology Assessment, National Health Commission of the People's Republic of China, Fudan University, Shanghai, China; ^4^Shanghai Ninth People's Hospital, Shanghai JiaoTong University School of Medicine, Shanghai, China; ^5^Department of Radiology, Shanghai Institute of Medical Imaging, Shanghai, China; ^6^Huashan Hospital, Fudan University, Shanghai, China; ^7^Department of Psychology, University of California, Los Angeles, Los Angeles, CA, United States; ^8^Curtin University of Technology, Perth, WA, Australia; ^9^Department of Management, University of California, Los Angeles, Los Angeles, CA, United States; ^10^Department of Epidemiology, University of California, Los Angeles, Los Angeles, CA, United States; ^11^Department of Epidemiology and Biostatistics, College for Public Health and Social Justice, Saint Louis University, St. Louis, MO, United States; ^12^Department of Finance, Shanghai Children's Hospital, Shanghai Jiao Tong University, Shanghai, China

**Keywords:** COVID-19, influenza A, differential diagnosis, rapid triage tools, regression tree analysis

## Abstract

**Background:** The coronavirus disease 2019 (COVID-19) pandemic has lasted much longer than an influenza season, but the main signs, symptoms, and some imaging findings are similar in COVID-19 and influenza patients. The aim of the current study was to construct an accurate and robust model for initial screening and differential diagnosis of COVID-19 and influenza A.

**Methods:** All patients in the study were diagnosed at Fuyang No. 2 People's Hospital, and they included 151 with COVID-19 and 155 with influenza A. The patients were randomly assigned to training set or a testing set at a 4:1 ratio. Predictor variables were selected based on importance, assessed by random forest algorithms, and analyzed to develop classification and regression tree models.

**Results:** In the optimal model A, the best single predictor of COVID-19 patients was a normal or high level of low-density lipoprotein cholesterol, followed by low level of creatine kinase, then the presence of <3 respiratory symptoms, then a highest temperature on the first day of admission <38°C. In the suboptimal model B, the best single predictor of COVID-19 was a low eosinophil count, then a normal monocyte ratio, then a normal hematocrit value, then a highest temperature on the first day of admission of <37°C, then a complete lack of respiratory symptoms.

**Conclusions:** The two models provide clinicians with a rapid triage tool. The optimal model can be used to developed countries/regions and major hospitals, and the suboptimal model can be used in underdeveloped regions and small hospitals.

## Introduction

On March 11, 2020, the World Health Organization (WHO) announced that coronavirus disease 2019 (COVID-19) caused by severe acute respiratory syndrome coronavirus 2 (SARS-CoV-2) infection had become a global pandemic (1, 2). With the growing number of SARS-CoV-2 infection and associated fatalities, the early diagnosis of COVID-19 has become a priority (3). A positive SARS-CoV-2 nucleic acid test is currently the gold standard for the diagnosis of COVID-19 (4–6), but nucleic acid testing is subject to false-negative and false-positive results (7, 8). Therefore, both the World Health Organization and National Health Commission of the People's Republic of China recommend comprehensive consideration of historical epidemiology, imaging results, clinical signs and symptoms, and laboratory evidence such as etiology or serology indicators for diagnosis (9). These methods are labor-intensive, however, and require substantial material and medical resources.

Seasonal influenza viruses can cause acute respiratory infection and a high rate of morbidity and mortality (10, 11). They are classified into four types: A, B, C, and D. Among them, H1N1 influenza A viruses are quite common and are associated with a high mortality rate, for example the H1N1 “swine flu” which caused an influenza pandemic in 2009 (11).

Distinguishing between influenza and COVID-19 can be problematic because their main signs and symptoms are similar (12, 13). Although some differentiation between COVID-19 and influenza patients is possible *via* chest computed tomography features, different radiologists, scanning parameters, image quality, and stages of disease may affect interpretations of certain imaging details (14–16).

Given that often only limited diagnostic and treatment resources are available, triage tools that enable rapid differential identification of COVID-19 and seasonal influenza are crucial to facilitate the allocation of appropriate medical resources and the application of prevention and control measures.

The current study included 151 COVID-19 patients and 155 patients with influenza A pneumonia from a hospital in Anhui Province in China. Based on symptoms (especially in the first 3 days), clinical signs, and physical and chemical laboratory test indicators a new model for the initial screening and differential diagnosis of COVID-19 and seasonal influenza pneumonia was constructed.

## Materials and Methods

### Patients

All patients were adults with COVID-19 or influenza A confirmed at the Fuyang No. 2 People's Hospital, Anhui Province, China. The study was approved by the Ethics Committee of the Fuyang No. 2 People's Hospital. The inclusion criteria were age equal and above 18 years, hospitalization with complete medical history, temperature records, complete blood count and serum biochemical indicators, and a confirmed diagnosis via SARS-CoV-2 or influenza A virus detection.

### Research Procedures and Data Collection

Respiratory tract samples including oropharyngeal swab, sputum, bronchial lavage, and blood and fecal specimens were obtained from COVID-19 patients at hospital admission, stored in viral transport medium, then sent to the Disease Control and Prevention Center of Fuyang for laboratory verification of SARS-CoV-2. Bilateral tonsils and posterior pharyngeal swabs collected from patients with influenza A were sent to the Influenza Surveillance Laboratory (National Influenza Surveillance Network Laboratory) of the Disease Control and Prevention Center of Fuyang for pathogen determination. The admission examination of patients included complete blood count, and blood biochemistry including renal function, liver function, creatine kinase (CK), lactate dehydrogenase, and electrolytes.

Nursing records and laboratory examination results of adults confirmed COVID-19 and influenza A patients at Fuyang No. 2 People's Hospital were retrospectively collated. Admission data from COVID-19 patients ranged from 20 January 2020 to 17 February 2020. Admission data from influenza A patients ranged from 08 April 2013 to 18 April 2019. A standardized data collection form was used to record patients' demographic characteristics, clinical symptoms, and laboratory results. COVID-19 patient data were acquired from the hospital's electronic medical records, whereas influenza A patient data were acquired from both printed and electronic medical records. All data were recorded and reviewed by 5 researchers to ensure that the data collected were authentic and valid.

### Laboratory Findings

Complete blood counts were acquired using an XE-2100 automatic hematology analyzer (Sysmex Corporation, Japan). Serum biochemical tests (including renal and liver function, CK, lactate dehydrogenase, and electrolytes), myocardial enzymes, and C-reactive protein were analyzed using a Hitachi 7180 automatic analyzer (Hitachi, Tokyo, Japan).

### Coronavirus and Influenza A Virus Testing

The local Center for Disease Control and Prevention performed SARS-CoV-2 detection in respiratory specimens by real-time fluorescent RT-PCR. The local Center for Disease Control and Prevention of Influenza surveillance laboratory (National influenza surveillance network laboratory) performed influenza A virus detection in pharyngeal swabs via RT-PCR methods with commercial assay kits provided by Beijing Kinghawk Pharmaceutical CO., Ltd. (Beijing, China).

### Modeling and Verification

Because reference ranges of some indicators in laboratory examination results vary with different kits and other factors, some results could not be directly compared, thus they were converted into the following groups of indicators: (1) Lower than the reference, (2) normal, (3) higher than the reference. Continuous variables are presented as medians and interquartile ranges (IQRs), and categorical variables are presented as numbers and percentages. Odds ratios (ORs) and confidence intervals (CIs) were calculated, and logistic regression was used to compare the ORs of each variable in COVID-19 and influenza A patients. The random forest method was then used to determine the influence weighting of each variable and the risk factors with the greatest effects. Based on the results of random forest analysis three variables were selected, respectively, from the three groups of indicators, i.e., (1) demographic characteristics, clinical signs, and symptoms, (2) routine blood results, and (3) serum biochemistry results. A classification and regression tree (CART) model was then used to construct a decision tree. A training set (245 patients) and a testing set (61 patients) were created based on a ratio of 4:1. The training set was used for modeling and the testing set was used for verification.

Areas under the curve (AUCs) and a confusion matrix were used to evaluate the efficiency and robustness of the established models. Based on the characteristics of the models, sensitivity and specificity were calculated using the testing set. All statistical analyses were performed using R (version 3.6.3) with a significance level of *p* < 0.05.

## Results

### Patient Characteristics

The demographic characteristics, clinical signs and symptoms, routine blood test results, and serum biochemistry results of the 306 patients included in the study are shown in Supplementary Table 1. There was no significant difference in age between the 151 COVID-19 patients (median 43 years, IQR 29–56 years) and the 155 influenza A patients (median 39 years, IQR 28–60). Men were at a 1.9 times greater risk of COVID-19 than women.

Both diseases tended to trigger fever at the onset of illness (94.2% of influenza A patients, 82.1% of COVID-19 patients), but the body temperature of influenza A patients on the first day of admission and the daily highest temperature in the first 3 days were higher than the corresponding medians in COVID-19 patients. COVID-19 patients were prone to diarrhea (OR 7.2, 95% CI 1.9–46.6), whereas influenza A patients showed more number of respiratory symptoms (OR 0.4, 95% CI 0.3–0.5); mainly coughing, expectoration, nasal discharge, pharyngalgia, chest congestion, and shortness of breath.

Complete blood count data on admission are shown in Supplementary Table 1. COVID-19 patients had lower white blood cell counts (OR 1.3, 95% CI 0.7–2.6), lymphocyte counts (OR 3.3, 95% CI 2.0–5.4), eosinophil counts (EO#s) (OR 79.0, 95% CI 28.2–330.3), and platelet counts (OR 5.4, 95% CI 2.2–16.4), and increased mean corpuscular hemoglobin concentration (OR 6.2, 95% CI 2.7–16.9).

Liver function remained normal in most patients, but elevated alanine aminotransferase (OR 2.5, 95% CI 1.2–5.5) and elevated aspartate aminotransferase (OR 2.6, 95% CI 1.4–5.3) were associated with a higher risk of COVID-19 infection. Increased low-density lipoprotein concentration was a protective factor in COVID-19 patients (OR 0.2, 95% CI 0.1–0.4). COVID-19 patients were more likely to exhibit abnormal cardiac enzymes than influenza A patients, as evidenced by a decrease in CK (OR 19.8, 95% CI 6.9–83.8) and an increase in lactate dehydrogenase (OR 1.7, 95% CI 1.1–2.7). Increased C-reactive protein concentration was evident in most patients (236/291, 81.1%), but it was more likely to be increased in influenza A patients (93.8%) than in COVID-19 patients (68.3%) (OR 0.1, 95% CI 0.0–0.3).

### Random Forest Ranking

**Figure 1:**
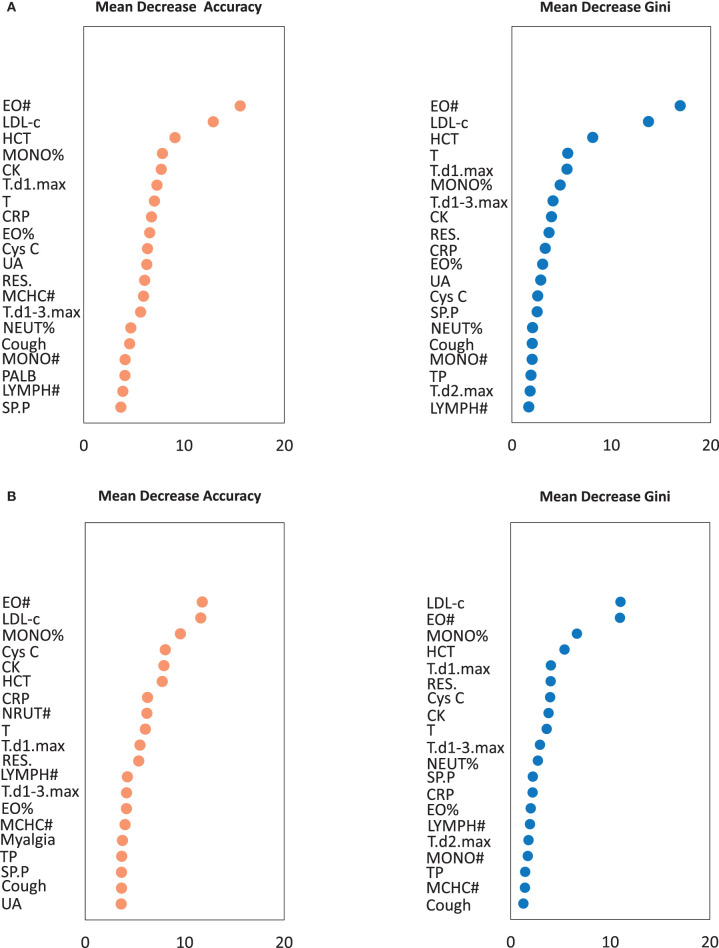
The importance of variables in the random forest algorithm. **(A)** The importance of variables was calculated by all patients. **(B)** The importance of variables was calculated by training set. A multi-indicator model was constructed by combining 61 variables (*P* < 0.05 in logistic regression analysis). Only Top 20 was shown. EO#, eosinophil count; LDL-c, Low-density lipoprotein cholesterol; HCT, Hematocrit; MONO%, monocyte ratio; CK, creatine kinase; T, Body temperature of the admission day; T.d1.max, Highest temperature on the first day of admission; T.d2.max, Highest temperature on the second day of admission; T.d1-3.max, Highest body temperature during the first 3 days of admission; RES., The number of respiratory symptoms; SP.P, Sputum production; CRP, C-reactive protein; EO%, Eosinophil ratio; MONO#, Monocyte count; MCHC**#**, Mean corpuscular hemoglobin concentration; NEUT%, Neutrophil ratio; LYMPH#, Lymphocyte count; Cys C, Cystatin C; UA, Uric acid; PALB, Prealbumin; TP, Total protein.

The results of random forest analysis are shown in Figure 1. The mean decrease accuracy plot and the mean decrease in Gini indicated that among clinical signs and symptoms, routine blood tests, and serum biochemistry results the most important variables were (1) highest temperature on the first day of admission, the number of respiratory symptoms, and coughing; (2) EO#, hematocrit, and monocyte ratio (MONO%); and (3) low-density lipoprotein cholesterol (LDL-c), C-reactive protein, and CK.

### CART Model

**Figure 2:**
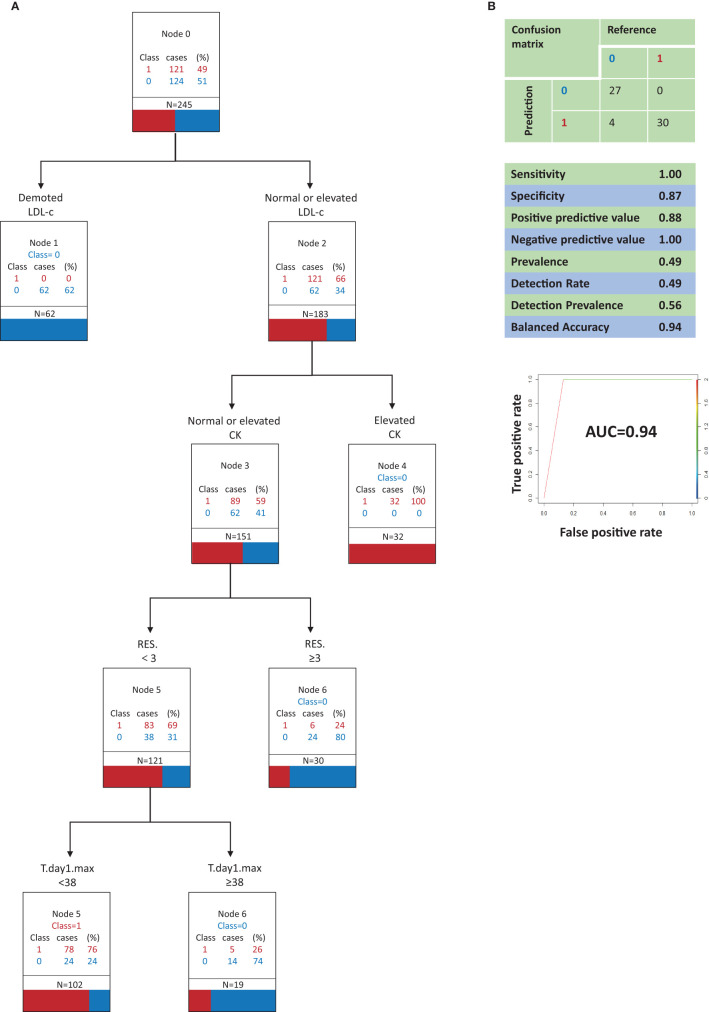
Classification and regression tree analysis of variables that most distinguish COVID-19 from Influenza A in clinical signs and symptoms and in serum biochemistry (model A, optimal model). **(A)** 0, Influenza A; 1, COVID-19; N, the total number of patients; RES., The number of respiratory symptoms; T.day1.max, Highest temperature on the first day of admission; LDL-c, Low-density lipoprotein cholesterol; CK, creatine kinase. All factors are compared with the limit of the range of medical reference value. **(B)** Performance characteristics of the model validated by the testing set.

**Figure 3:**
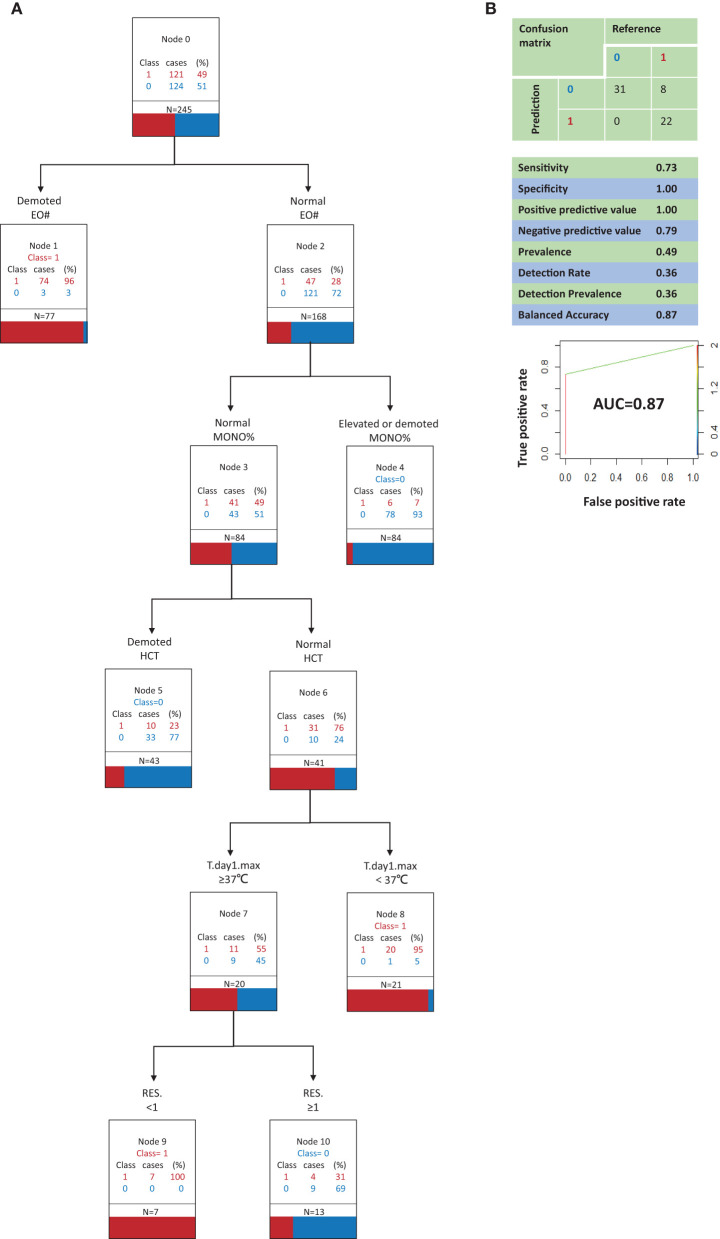
Classification and regression tree analysis of variables that most distinguish COVID-19 from Influenza A in clinical signs and symptoms and in routine blood (model B, suboptimal model). **(A)** 0, Influenza A; 1, COVID-19; *N*, the total number of patients; RES., The number of respiratory symptoms; T.day1.max, Highest temperature on the first day of admission; EO#, eosinophil count; MONO%, monocyte ratio; HCT, Hematocrit. All factors are compared with the limit of the range of medical reference value. **(B)** Performance characteristics of the model validated by the testing set.

The distributions of 9 important variables identified in the training set and the testing set were similar in the random forest plots (Table 1). CART modeling was then used to construct decision models (model A, Figure 2; model B, Figure 3) of clinical signs and symptoms and serum biochemistry, and clinical signs and symptoms and routine blood results of the 245 patients in the training set. Decision-making models C, D, and E were generated separately for the aforementioned three types of indicators, and an overall decision-making model (model F) was generated.

**Table 1:** Characteristics of factors included in CART modeling, between training set and testing set.

	**Training**						**Testing**					
	**Influenza A (*n* = 124)**	**COVID-19 (*n* = 121)**	**All patients (*n* = 245)**	**Coef**.	**OR (95% CI)**	***P***	**Influenza A (*n* = 31)**	**COVID-19 (*n* = 30)**	**All patients (*n* = 61)**	**Coef**.	**OR (95% CI)**	***P***
The number of respiratory symptoms	2 (2, 3)	1 (0, 2)	2 (1, 3)	−1.1	0.3 (0.2, 0.5)	<0.001	2 (2, 3)	1 (0, 2)	2 (1, 2)	−1.0	0.4 (0.2, 0.7)	0.0029
Highest temperature on the first day of admission	37.9 (37.0, 38.5)	37.0 (36.7, 37.7)	37.3 (36.8, 38.0)	−0.8	0.4 (0.3, 0.6)	<0.001	38.3 (37.4, 38.7)	37.0 (36.6, 37.4)	37.4 (36.8, 38.3)	−1.8	0.2 (0.1, 0.4)	<0.001
Missing	1 (0.8%)	0 (0.0%)	1 (0.4%)									
Cough	111 (89.5%)	75 (62.0%)	186 (75.9%)	−1.7	0.2 (0.1, 0.4)	<0.001	28 (90.3%)	18 (60.0%)	46 (75.4%)	−1.8	0.2 (0.0, 0.6)	0.010
Eosinophil count, × 10^9^ per L	0.0 (0.0, 0.0)	0.0 (0.0, 0.0)	0.0 (0.0, 0.0)				0.0 (0.0, 0.0)	0.0 (0.0, 0.0)	0.0 (0.0, 0.0)			
Normal	121 (97.6%)	47 (38.8%)	168 (68.6%)				31 (100%)	12 (40.0%)	43 (70.5%)			
Lower	3 (2.4%)	74 (61.2%)	77 (31.4%)	4.2	63.5 (22.2, 268.1)	<0.001	0 (0.0%)	18 (60.0%)	18 (29.5%)	20.5	812112207.5 (0.0, NA)	0.99
Hematocrit, %	36.8 (33.1, 40.1)	40.3 (37.7, 43.0)	38.8 (35.4, 42.1)				36.4 (29.4, 39.0)	41.8 (38.5, 44.0)	38.6 (34.8, 42.7)			
Normal	34 (27.4%)	92 (76.0%)	126 (51.4%)				7 (22.6%)	22 (73.3%)	29 (47.5%)			
Lower	89 (71.8%)	28 (23.1%)	117 (47.8%)	−2.2	0.1 (0.1, 0.2)	<0.001	24 (77.4%)	6 (20.0%)	30 (49.2%)	−2.5	0.1 (0.0, 0.2)	<0.001
Higher	1 (0.8%)	1 (0.8%)	2 (0.8%)	−1.0	0.4 (0.0, 9.5)	0.49	0 (0.0%)	2 (6.7%)	2 (3.3%)	15.4	4979978.4 (0.0, NA)	0.99
Monocyte ratio, %	8.9 (5.3, 13.3)	6.9 (5.5, 9.3)	7.5 (5.4, 11.9)				9.35 (6.10, 12.7)	7.60 (6.38, 9.75)	8.40 (6.28, 10.9)			
Normal	45 (36.3%)	99 (81.8%)	144 (58.8%)				12/30 (40.0%)	22 (73.3%)	34/60 (56.7%)			
Lower	12 (9.7%)	0 (0.0%)	12 (4.9%)	−17.4	0.0 (NA, Inf)	0.98	2/30 (6.7%)	1 (3.3%)	3/60 (5.0%)	−1.3	0.3 (0.0, 3.1)	0.31
Higher	67 (54.0%)	22 (18.2%)	89 (36.3%)	−1.9	0.1 (0.1, 0.2)	<0.001	16/30 (53.3%)	7 (23.3%)	23/60 (38.3%)	−1.4	0.2 (0.1, 0.7)	0.013
Low-density lipoprotein cholesterol, mmol/L	2.1 (1.6, 2.7)	2.1 (1.7, 2.8)	2.1 (1.7, 2.8)				1.71 (1.47, 2.12)	2.13 (1.91, 2.46)	2.01 (1.64, 2.41)			
Normal	44/120 (36.7%)	99/110 (90.0%)	143/230 (62.2%)				3/27 (11.1%)	28/28 (100%)	31/55 (56.4%)			
Lower	60/120 (50.0%)	0/110 (0%)	60/230 (26.1%)	−1.2	0.3 (0.1, 0.7)	0.0060	20/27 (74.1%)	0 (0.0%)	20/55 (36.4%)	−22.8	0.0 (NA, Inf)	1.00
Higher	16/120 (13.3%)	11/110 (10.0%)	27/230 (11.7%)	−19.4	0.0 (0.0, 477716.3)	0.98	4/27 (14.8%)	0 (0.0%)	4/55 (7.3%)	−22.8	0.0 (NA, Inf)	1.00
C-reactive protein, mg/L	39.1 (18.9, 77.1)	13.2 (3.2, 35.6)	24.4 (7.95, 54.9)				37.0 (22.7, 59.3)	17.3 (3.23, 36.9)	23.8 (10.5, 57.5)			
Normal	8/118 (6.8%)	38/117 (32.5%)	46/235 (19.6%)				1/28 (3.6%)	8/28 (28.6%)	9/56 (16.1%)			
Higher	110/118 (93.2%)	79/117 (67.5%)	189/235 (80.4%)	−1.9	0.2 (0.1, 0.3)	<0.001	27/28 (96.4%)	20/28 (71.4%)	47/56 (83.9%)	−2.4	0.1 (0.0, 0.6)	0.031
Creatine kinase, U/L	75.0 (46.0, 134.0)	62.0 (47.0, 88.0)	69.0 (46.5, 107.0)				63.5 (43.8, 156)	58.0 (38.8, 88.3)	58.0 (40.0, 123)			
Normal	105 (84.7%)	68/103 (66.0%)	173/227 (76.2%)				23/28 (82.1%)	16 (72.7%)	39 (75.0%)			
Lower	3 (2.4%)	32/103 (31.1%)	35/227 (15.4%)	2.8	16.5 (5.6, 70.4)	<0.001	0/28 (0.0%)	7 (31.8%)	7 (13.5%)	17.9	61158167.7 (0.0, NA)	0.99
Higher	16 (12.9%)	3/103 (2.9%)	19/227 (8.4%)	−1.2	0.3 (0.1, 0.9)	0.056	5/28 (17.9%)	1 (4.5%)	6 (11.5%)	−1.2	0.3 (0.0, 2.0)	0.28

*Data are median (IQR), n (%), or n/N (%), where N is the total number of patients with available data. Comparing COVID-19 and influenza A are from logistic regression*.

*Coef., coefficient; OR, odds ratio; CI, confidence interval; Inf, infinity*.

Figure 2A depicts decision tree model A constructed with 6 clinical signs and symptoms and serum biochemistry variables. The best indicator for distinguishing COVID-19 patients from influenza A patients was a decrease in LDL-c, which was associated with influenza A. When LDL-c was normal or elevated, a decrease in the secondary indicator CK contributed to the ability to identify COVID-19 patients. When LDL-c was normal or elevated and CK was normal or elevated, the third most important indicator was the number of respiratory symptoms present. When LDL-c was normal or elevated and CK was normal or elevated, the presence of ≥3 respiratory symptoms contributed to the ability to identify influenza A patients. When LDL-c was normal or elevated, CK was normal or elevated, and there were <3 respiratory symptoms, the highest body temperature on the first day of admission was the fourth most important indicator. When LDL-c was normal or elevated, CK was normal or elevated, and there were <3 respiratory symptoms, a highest temperature on the first day of admission of <38°C contributed to the ability to identify COVID-19 patients, whereas a highest body temperature of ≥38°C on the first day of admission contributed to the ability to identify influenza A patients.

Figure 3A depicts decision tree model B constructed with 6 clinical signs and symptoms and routine blood test variables. The best indicator for distinguishing COVID-19 patients from influenza A patients was a low EO#. When the EO# was normal, an increase or decrease in the MONO% contributed to the ability to identify influenza A patients. When the EO# and MONO% were both normal, the third most important indicator was hematocrit (HCT). When the EO# and MONO% were both normal, low HCT contributed to the ability to identify influenza A patients. When the EO#, MONO%, and HCT were all normal, the fourth most important indicator was the highest body temperature on the first day of admission. When the EO#, MONO%, and HCT were all normal, a highest body temperature of <37°C on the first day of admission contributed to the ability to identify COVID-19 patients. When the EO#, MONO%, and HCT were all normal and the highest body temperature on the first day of admission was ≥37°C, the fifth most important indicator was the number of respiratory symptoms present. When the EO#, MONO%, and hematocrit were all normal and the highest temperature on the first day of admission was ≥37°C, a complete lack of respiratory symptoms contributed to the ability to identify COVID-19 patients, whereas the presence of ≥1 respiratory symptom contributed to the ability to identify influenza A patients.

### Model Validation

Model A (Figure 2B) correctly identified all COVID-19 patients, but it also incorrectly classified 4 influenza A patients as COVID-19 patients. Model A had a sensitivity of 1.00, a specificity of 0.87, a positive predictive value of 0.88, and a negative predictive value of 1.00. In a receiver operating characteristic curve of model A the AUC was 0.93.

Model B (Figure 3B) correctly identified all influenza A patients, but it also incorrectly classified 8 COVID-19 patients as influenza A patients. Model B had a sensitivity of 0.73, a specificity of 1.00, a positive predictive value of 1.00, and a negative predictive value of 0.79. In a receiver operating characteristic curve of model B the AUC was 0.87.

Confusion matrix analysis indicating the difference between the prediction results generated by models C–F and the real results among the testing set patients is shown in Supplementary Figures 2–5.

Model A demonstrated the best predictive capacity with respect to both COVID-19 patients and influenza A patients. Especially when it is necessary to predict and identify a COVID-19 patient, model A is able to minimize misdiagnosis and thus it is considered the optimal model. Model B also exhibited a favorable predictive performance in COVID-19 patients and influenza A patients, so it is regarded as a valid albeit suboptimal model.

## Discussion

In the current study differential diagnosis models of COVID-19 and influenza A patients were generated based on individual signs and symptoms, routine blood tests, and serum biochemistry results. The models were classified as optimal (model A) or suboptimal (model B) with respect to their capacity for differential diagnosis. Given that the routine blood testing required in the suboptimal model is a more economical and common laboratory tool, that model is more suitable for underdeveloped areas. Both models A and B were accurate, sensitive, and robust, so that they can offer technical support for rapid clinical triage.

In the optimal model the frequency of respiratory symptoms and the highest temperature on the first day of admission were included as indicators. Patients with a greater number of respiratory symptoms were more likely to have influenza A than COVID-19. This is consistent with previous reports indicating that respiratory symptoms—especially upper respiratory symptoms—are not substantial in many COVID-19 patients (17). A high temperature on the first day of admission also indicated that influenza A was more likely than COVID-19. This is concordant with previous studies in which influenza A patients generally had high fever at the onset of illness (18, 19), whereas many COVID-19 patients exhibit no initial symptoms such as high fever (20).

With regard to routine blood tests, EO#, MONO%, and HCT were incorporated into model B (signs and symptoms + routine blood tests). Previous studies have not suggested changes or abnormalities in these three indicators in patients with influenza A or COVID-19 (21–25). However, in the present study model B indicated that combined with signs and symptoms and routine blood results, these three indicators can be used to distinguish between influenza A patients and COVID-19 patients. The pathophysiological basis underlying differences in these indicators in the two groups of patients warrants further research.

Although the incidence of decreased LDL-c did not differ significantly between COVID-19 patients and influenza A patients, most influenza A patients exhibited decreased LDL-c whereas no COVID-19 patients did. This indicator was well-distinguished in a subsequently generated CART algorithm. Lastly, in model A LDL-c was the most important indicator. It has previously been reported that C-reactive protein and CK may be elevated in influenza A patients (18, 26). Most COVID-19 patients have exhibited elevated C-reactive protein, and a few of them have exhibited elevated CK (22, 25, 27). In the current study model A indicated that increased CK suggested that influenza A was more likely than COVID-19, which was the same as previous studies (22, 28–30). C-reactive protein was excluded as an unimportant indicator. Although there is evidence that COVID-19 may lead to complications of heart disease (29), in the present study normal or reduced CK was more suggestive of COVID-19 than influenza A.

The initial classification model was not completely consistent with the preferred indicators applied alone for the diagnosis of COVID-19 or influenza A. According to the COVID-19 diagnosis and treatment plan published in China, in addition to a history of potential exposure the determination of suspected cases mainly involved the total number of white blood cells and lymphocyte counts with respect to clinical symptoms and laboratory examinations (12, 23, 28). Because the primary aim of the current study was to distinguish between influenza A and COVID-19 patients, there were differences between the laboratory indicators and the combinations of them used in the constructed models, and the indicators emphasized in the treatment plan. Notably, the model developed in the present study was designed to distinguish between patients with suspected influenza A or COVID-19 rather than simply identify COVID-19 patients.

The prevalence of COVID-19 in children is very low, and in one study a prevalence of just 2.1% among a group of people aged 0–18 years was reported (29). Conversely the prevalence of influenza A in children is higher, and can reportedly reach 25.7% (30). Therefore, inclusion of children in the current study could have introduced mixed effects caused by age. For this reason people under the age of 18 were excluded, and the diagnostic model tool was also constructed for adults. Diagnostic tools and models specifically designed for use in children can be developed in the future.

The current study had some limitations. The sample size was small. Although the models constructed were sensitive and robust, large numbers of COVID-19 and influenza A cases should be used in the future to further verify and develop the models. Another limitation was that due to yearly changes in influenza viral antigenic configuration, the conditions of historical cases may differ from those of current cases. With respect to influenza strains, the present study only involved H1N1. Lastly, the lack of anosmia data may affect the differentiation capacity of the model.

In the current study an optimal model for distinguishing between influenza A and COVID-19 patients was generated. Another tool for initial screening and identification based on individual signs and symptoms and routine blood indicators was also generated for use in underdeveloped areas where the economy, detection capacity, and medical resources may not be conducive to blood biochemistry examinations. In developing countries such as China, the cost of a routine blood test is only 1/6 of that of blood biochemistry examination, and in less developed regions it can cost merely 1/10. Therefore, a simplified identification tool is of high cost-benefit value, although it reduces the ability to identify COVID-19 patients, which may inevitably lead to a degree of misdiagnosis.

## Data Availability Statement

The raw data supporting the conclusions of this article will be made available by the authors, without undue reservation.

## Ethics Statement

The studies involving human participants were reviewed and approved by Ethics Committee of the Fuyang No. 2 People's Hospital, Anhui Province. The patients/participants provided their written informed consent to participate in this study. Written informed consent was obtained from the individual(s) for the publication of any potentially identifiable images or data included in this article.

## Author Contributions

BY and WZhe had the idea for and designed the study and had full access to all data in the study and take responsibility for the integrity of the data and the accuracy of the data analysis. AX collected the data of COVID-19 and human influenza patients. AX and HZ were in charge of the manuscript draft. AX, HZ, JX, LZ, KZ, BY, and WZhe contributed to writing the report. HZ, JX, CZ, ZR, NM, and XL contributed to data input, cleaning, and database establishment. HZ, LZ, WM, WZhu, and DT contributed to the statistical analysis. ZW, YH, and JL contributed to the data and results checking, review and revised the manuscript. All authors contributed to data acquisition, data analysis, or data interpretation, and reviewed and approved the final version.

## Conflict of Interest

The authors declare that the research was conducted in the absence of any commercial or financial relationships that could be construed as a potential conflict of interest.

## Publisher's Note

All claims expressed in this article are solely those of the authors and do not necessarily represent those of their affiliated organizations, or those of the publisher, the editors and the reviewers. Any product that may be evaluated in this article, or claim that may be made by its manufacturer, is not guaranteed or endorsed by the publisher.
