# Supplementary material for: Triage Modeling for Differential Diagnosis Between COVID-19 and Human Influenza A Pneumonia: Classification and Regression Tree Analysis
Source: Front Med (Lausanne). 2021 Aug 10;8:673253. doi: 10.3389/fmed.2021.673253 (PMC8382719; doi:10.3389/fmed.2021.673253)
Supplement: Supplementary file 1 [file Table_1.docx]

| **Table S1: Demographics and baseline characteristics of patients infected with COVID-19 and influenza A** | | | | | | | | | | | | | | |
| --- | --- | --- | --- | --- | --- | --- | --- | --- | --- | --- | --- | --- | --- | --- |
|  | | **All patients**  **(n=306)** | | **Influenza A**  **(n=155)** | **COVID-19**  **(n=151)** | | **Coef.** | | **OR [95%CI]** | | | | | **P** |
| **Characteristics** | | | | | | | | | | | | | | |
| Age, years | | 43.0 [29.0,56.0] | | 39.0 [28.0,60.0] | 46.0 [36.0,53.5] | | 0.0 | | 1.0 [1.0,1.0] | | | | | 0.82 |
| Sex | |  | |  |  | |  | |  | | | | |  |
| Female | | 158 (51.6%) | | 92 (59.4%) | 66 (43.7%) | |  | |  | | | | |  |
| Male | | 148 (48.4%) | | 63 (40.6%) | 85 (56.3%) | | 0.6 | | 1.9 [1.2,3.0] | | | | | 0.0064 |
| Drinking | |  | |  |  | |  | |  | | | | |  |
| No | | 255/302(84.4%) | | 139 (89.7%) | 116 /147(78.9%) | |  | |  | | | | |  |
| Yes | | 37/302(12.3%) | | 14 (9.0%) | 23 /147(15.6%) | | 0.7 | | 2.0 [1,4.1.0] | | | | | 0.061 |
| Give up drinking | | 10/302(3.3%) | | 2 (1.3%) | 8/147 (5.4%) | | 1.6 | | 4.8 [1.2,32.2] | | | | | 0.050 |
| Smoking | |  | |  |  | |  | |  | | | | |  |
| No | | 256/302 (84.8%) | | 131 (84.5%) | 125/147 (85.0%) | |  | |  | | | | |  |
| Yes | | 25/302 (8.3%) | | 14 (9.0%) | 11/147 (7.5%) | | -0.2 | | 0.8 [0.4,1.9] | | | | | 0.65 |
| Give up smoking | | 21/302 (7.0%) | | 10 (6.5%) | 11 /147(7.5%) | | 0.1 | | 1.2 [0.5,2.9] | | | | | 0.75 |
| Married | | 265/303 (87.5%) | | 134 (86.5%) | 131 /148(88.5%) | | 0.2 | | 1.2 [0.6,2.4] | | | | | 0.59 |
| **Signs and symptoms** | | | | | | | | | | | | | | |
| The number of respiratory symptoms | | 2 [1,2] | | 2 [2,3] | 1 [0,2] | | -1.0 | | 0.4 [0.3,0.5] | | | | | <0.001 |
| Fever | | 270 (88.2%) | | 146 (94.2%) | 124 (82.1%) | | -1.3 | | 0.3 [0.1,0.6] | | | | | 0.0018 |
| Body temperature of the admission day | | 37.1 [36.7,37.9] | | 37.5 [36.8,38.3] | 36.9 [36.6,37.4] | | -0.9 | | 0.4 [0.3,0.6] | | | | | <0.001 |
| Highest temperature on the first day of admission | | 37.4 [36.8,38.2] | | 37.9 [37.0,38.5] | 37.0 [36.7,37.6] | | -1.0 | | 0.4 [0.3,0.5] | | | | | <0.001 |
|  | | | | | | | | | | | | | | |
| (Continued from previous page) | | | | | | | | | | | | | | |
|  | | **All patients**  **(n=306)** | | **Influenza A**  **(n=155)** | **COVID-19**  **(n=151)** | | **Coef.** | | **OR [95%CI]** | | | | | **P** |
| Missing | | 1 (0.3%) | | 1 (0.6%) | 0 (0%) | |  | |  | | | | |  |
| Highest temperature on the second day of admission | | 37.0 [36.8,37.6] | | 37.1 [36.8,37.8] | 37.0 [36.8,37.5] | | -0.5 | | 0.6 [0.4,0.9] | | | | | 0.0086 |
| Missing | | 2 (0.7%) | | 2 (1.3%) | 0 (0%) | |  | |  | | | | |  |
| Highest temperature on the third day of admission | | 36.9 [36.8,37.3] | | 37.0 [36.8,37.2] | 36.9 [36.7,37.4] | | 0.2 | | 1.3 [0.9,1.9] | | | | | 0.24 |
| Missing | | 7 (2.3%) | | 7 (4.5%) | 0 (0%) | |  | |  | | | | |  |
| Highest temperature during the first three days of admission | | 37.8 [37.0,38.5] | | 38.1 [37.3,38.6] | 37.3 [37.0,38.0] | | -0.9 | | 0.4 [0.3,0.6] | | | | | <0.001 |
| Coughing | | 232 (75.8%) | | 139 (89.7%) | 93 (61.6%) | | -1.7 | | 0.2 [0.1,0.3] | | | | | <0.001 |
| Dyspnea | | 4 (1.3%) | | 3 (1.9%) | 1 (0.7%) | | -1.1 | | 0.3 [0.0,2.7] | | | | | 0.35 |
| Pharyngalgia | | 49 (16.0%) | | 35 (22.6%) | 14 (9.3%) | | -1.0 | | 0.4 [0.2,0.7] | | | | | 0.0020 |
| Shortness of breath | | 44 (14.4%) | | 34 (21.9%) | 10 (6.6%) | | -1.4 | | 0.3 [0.1,0.5] | | | | | <0.001 |
| Xerostomia | | 6 (2.0%) | | 0 (0%) | 6 (4.0%) | | 15.6 | | 6154903.4 [0.0, NA] | | | | | 0.98 |
| Chest congestion | | 73 (23.9%) | | 47 (30.3%) | 26 (17.2%) | | -0.7 | | 0.5 [0.3,0.8] | | | | | 0.0078 |
| Stethalgia | | 3 (1.0%) | | 1 (0.6%) | 2 (1.3%) | | 0.7 | | 2.1 [0.2,44.8] | | | | | 0.56 |
| Dizzy | | 15 (4.9%) | | 10 (6.5%) | 5 (3.3%) | | -0.7 | | 0.5 [0.2,1.4] | | | | | 0.21 |
| Nasal obstruction | | 4 (1.3%) | | 1 (0.6%) | 3 (2.0%) | | 1.1 | | 3.1 [0.4,63.5] | | | | | 0.33 |
| Nasal discharge | | 28 (9.2%) | | 26 (16.8%) | 2 (1.3%) | | -2.7 | | 0.1 [0.0,0.2] | | | | | <0.001 |
| Pharyngeal itching | | 1 (0.3%) | | 0 (0%) | 1 (0.7%) | | 14.6 | | 2188786 [0.0, NA] | | | | | 0.99 |
| Myalgia | | 30 (9.8%) | | 25 (16.1%) | 5 (3.3%) | | -1.7 | | 0.2 [0.1,0.4] | | | | | 0.00063 |
|  | | | | | | | | | | | | | | |
| (Continued from previous page) | | | | | | | | | | | | | | |
|  | **All patients**  **(n=306)** | | **Influenza A**  **(n=155)** | | | **COVID-19**  **(n=151)** | | **Coef.** | | | | **OR [95%CI]** | **P** | |
| Expectoration | 168 (54.9%) | | 112 (72.3%) | | | 56 (37.1%) | | -1.5 | | | | 0.2 [0.1,0.4] | <0.001 | |
| Headache | 28 (9.2%) | | 21 (13.5%) | | | 7 (4.6%) | | -1.2 | | | | 0.3 [0.1,0.7] | 0.0097 | |
| Diarrhoea | 15 (4.9%) | | 2 (1.3%) | | | 13 (8.6%) | | 2.0 | | | | 7.2 [1.9,46.6] | 0.010 | |
| Fatigue | 44 (14.4%) | | 25 (16.1%) | | | 19 (12.6%) | | -0.3 | | | | 0.7 [0.4,1.4] | 0.38 | |
| Chills | 22 (7.2%) | | 13 (8.4%) | | | 9 (6.0%) | | -0.4 | | | | 0.7 [0.3,1.7] | 0.41 | |
| Nausea or vomiting | 21 (6.9%) | | 14 (9.0%) | | | 7 (4.6%) | | -0.7 | | | | 0.5 [0.2,1.2] | 0.14 | |
| Anorexia | 17 (5.6%) | | 6 (3.9%) | | | 11 (7.3%) | | 0.7 | | | | 2 [0.7,5.8] | 0.20 | |
| Poor mental, diet or sleep | 119 (38.9%) | | 115 (74.2%) | | | 4 (2.6%) | | -4.7 | | | | 0 [0.0,0.0] | <0.001 | |
| Systolic blood pressure, mmHg | 124.0 [115.0,138.0] | | 119.0 [110.0,131.0] | | | 129.0 [120.0,141.0] | | 0.0 | | | | 1 [1.0,1.1] | <0.001 | |
| Diastolic blood pressure, mmHg | 80.0 [72.0,89.0] | | 76.0 [69.0,84.0] | | | 85.0 [77.0,92.5] | | 0.1 | | | | 1.1 [1.0,1.1] | <0.001 | |
| **Laboratory findings** | | | | | | | | | | | | | | |
| White blood cell count, × 10^9^ per L | 5.7 [4.3,8.0] | | 6.9 [5.1,8.9] | | | 5.0 [3.9,6.4] | |  | |  | | |  | |
| Normal | 230 (75.2%) | | 109 (70.3%) | | | 121 (80.1%) | |  | |  | | |  | |
| Lower | 42 (13.7%) | | 17 (11.0%) | | | 25 (16.6%) | | 0.3 | | | | 1.3 [0.7,2.6] | 0.41 | |
| Higher | 34 (11.1%) | | 29 (18.7%) | | | 5 (3.3%) | | -1.9 | | | | 0.2 [0.1,0.4] | <0.001 | |
| Neutrophil ratio, % | 71.4 [61.5,80.1] | | 73.2 [64.9,81.8] | | | 70.6 [61.0,77.6] | |  | | | |  |  | |
| Normal | 145 (47.4%) | | 47 (30.3%) | | | 98 (64.9%) | |  | | | |  |  | |
| Lower | 20 (6.5%) | | 18 (11.6%) | | | 2 (1.3%) | | -2.9 | | | | 0.1 [0,0.2] | <0.001 | |
| Higher | 141 (46.1%) | | 90 (58.1%) | | | 51 (33.8%) | | -1.3 | | | | 0.3 [0.2,0.4] | <0.001 | |
| lymphocyte ratio, % | 18.2 [12.6,25.9] | | 15.4 [10.8,23.3] | | | 20.8 [15.3,29.5] | |  | | | |  |  | |
| Normal | 130 (42.5%) | | 50 (32.3%) | | | 80 (53.0%) | |  | | | |  |  | |
|  |  | |  | | |  | |  | |  | | |  | |
|  |  | |  | | |  | |  | |  | | |  | |
| (Continued from previous page) | | | | | | | | | | | | | | |
|  | **All patients**  **(n=306)** | | **Influenza A**  **(n=155)** | | | **COVID-19**  **(n=151)** | | **Coef.** | | | **OR [95%CI]** | | **P** | |
| Lower | 169 (55.2%) | | 100 (64.5%) | | | 69 (45.7%) | | -0.8 | | | 0.4 [0.3,0.7] | | <0.001 | |
| Higher | 7 (2.3%) | | 5 (3.2%) | | | 2 (1.3%) | | -1.4 | | | 0.3 [0.0,1.2] | | 0.11 | |
| Monocyte ratio, % | 7.7 [5.50,11.8] | | 9.2 [5.4,13.2] | | | 7.1 [5.7,9.3] | |  | | |  | |  | |
| Normal | 178/305 (58.4%) | | 57/154 (37.0%) | | | 121 (80.1%) | |  | | |  | |  | |
| Lower | 15/305 (4.9%) | | 14/154 (9.1%) | | | 1 (0.7%) | | -3.4 | | | 0.0 [0.0,0.2] | | 0.0012 | |
| Higher | 112/305 (36.7%) | | 83/154 (53.9%) | | | 29 (19.2%) | | -1.8 | | | 0.2 [0.1,0.3] | | <0.001 | |
| Eosinophil ratio, % | 0.2 [0.0,0.4] | | 0.1 [0.0,0.4] | | | 0.2 [0.0,0.5] | |  | | |  | |  | |
| Normal | 65/305 (21.3%) | | 13/154 (8.4%) | | | 52 (34.4%) | |  | | |  | |  | |
| Lower | 237/305 (77.7%) | | 139/154 (90.3%) | | | 98 (64.9%) | | -1.7 | | | 0.2 [0.1,0.3] | | <0.001 | |
| Higher | 3/305 (1.0%) | | 2/154 (1.3%) | | | 1 (0.7%) | | -2.1 | | | 0.1 [0,1.4.0] | | 0.10 | |
| Basophil ratio, % | 0.0 [0.0,0.1] | | 0.1 [0.0,0.2] | | | 0.0 [0.0,0.1] | |  | | |  | |  | |
| Normal | 303/305 (99.3%) | | 152/154 (98.7%) | | | 151 (100%) | |  | | |  | |  | |
| Higher | 2/305 (0.7%) | | 2/154 (1.3%) | | | 0 (0%) | | -14.6 | | | 0.0 [NA,1.71302874183301E+36] | | 0.98 | |
| Neutrophil count, × 10⁹ per L | 4.0 [2.7,5.9] | | 5.0 [3.1,7.0] | | | 3.6 [2.5,4.7] | |  | | |  | |  | |
| Normal | 237 (77.5%) | | 110 (71.0%) | | | 127 (84.1%) | |  | | |  | |  | |
| Lower | 25 (8.2%) | | 14 (9.0%) | | | 11 (7.3%) | | -0.4 | | | 0.7 [0.3,1.6] | | 0.36 | |
| Higher | 44 (14.4%) | | 31 (20.0%) | | | 13 (8.6%) | | -1.0 | | | 0.4 [0.2,0.7] | | 0.0044 | |
| Lymphocyte count, × 10⁹ per L | 1.1 [0.8,1.4] | | 1.2 [0.8,1.5] | | | 1.1 [0.8,1.4] | |  | | |  | |  | |
| Normal | 192 /305(63.0%) | | 117 /154(76.0%) | | | 75 (49.7%) | |  | | |  | |  | |
|  |  | |  | | |  | |  | | |  | |  | |
|  |  | |  | | |  | |  | | |  | |  | |
| (Continued from previous page) | | | | | | | | | | | | | | |
|  | **All patients**  **(n=306)** | | **Influenza A**  **(n=155)** | | | **COVID-19**  **(n=151)** | | **Coef.** | | **OR [95%CI]** | | | **P** | |
| Lower | 112/305 (36.7%) | | 36 /154(23.4%) | | | 76 (50.3%) | | 1.2 | | 3.3 [2.0,5.4] | | | <0.001 | |
| Higher | 1/305 (0.3%) | | 1 /154(0.6%) | | | 0 (0%) | | -14.1 | | 0.0 [NA,9.90498524067352E+71] | | | 0.99 | |
| Monocyte count, × 10⁹ per L | 0.5 [0.3,0.7] | | 0.6 [0.4,0.8] | | | 0.4 [0.3,0.5] | |  | |  | | |  | |
| Normal | 227/305 (74.4%) | | 93/154 (60.4%) | | | 134 (88.7%) | |  | |  | | |  | |
| Lower | 14 /305(4.6%) | | 14/154 (9.1%) | | | 0 (0%) | | -16.9 | | 0.0 [NA,204631124402.6] | | | 0.98 | |
| Higher | 64 /305(21.0%) | | 47/154 (30.5%) | | | 17 (11.3%) | | -1.4 | | 0.3 [0.1,0.5] | | | <0.001 | |
| Eosinophil count, × 10⁹ per L | 0.0 [0.0,0.0] | | 0.0 [0.0,0.0] | | | 0.0[0.0,0.0] | |  | |  | | |  | |
| Normal | 211 (69.0%) | | 152 (98.1%) | | | 59 (39.1%) | |  | |  | | |  | |
| Lower | 95 (31.0%) | | 3 (1.9%) | | | 92 (60.9%) | | 4.4 | | 79.0 [28.2,330.3] | | | <0.001 | |
| Higher | 0 (0%) | | 0 (0%) | | | 0 (0%) | |  | |  | | |  | |
| Basophil count, × 10⁹ per L | 0.0 [0.00,0.0] | | 0.0 [0.0,0.0] | | | 0.0 [0.0,0.0] | |  | |  | | |  | |
| Normal | 303 (99.3%) | | 152 (98.7%) | | | 151 (100%) | |  | |  | | |  | |
| Higher | 2 (0.7%) | | 2 (1.3%) | | | 0 (0%) | | -14.6 | | 0.0 [NA,1.71302874183301E+36] | | | 0.98 | |
| Red blood cell count, × 10^12^ per L | 4.3 [4.0,4.7] | | 4.1 [3.8,4.5] | | | 4.5 [4.2,4.8] | |  | |  | | |  | |
| Normal | 265 (86.6%) | | 127 (81.9%) | | | 138 (91.4%) | |  | |  | | |  | |
|  |  | |  | | |  | |  | |  | | |  | |
|  |  | |  | | |  | |  | |  | | |  | |
| (Continued from previous page) | | | | | | | | | | | | | | |
|  | **All patients**  **(n=306)** | | **Influenza A**  **(n=155)** | | | **COVID-19**  **(n=151)** | | **Coef.** | | **OR [95%CI]** | | | **P** | |
| Lower | 37 (12.1%) | | 25 (16.1%) | | | 12 (7.9%) | | -0.8 | | 0.4 [0.2,0.9] | | | 0.028 | |
| Higher | 4 (1.3%) | | 3 (1.9%) | | | 1 (0.7%) | | -1.2 | | 0.3 [0.0,2.4] | | | 0.31 | |
| Hemoglobin, g/L | 131.0 [117.0,146.0] | | 121.0 [109.0,135.0] | | | 141.0 [129.0,151.0] | |  | |  | | |  | |
| Normal | 241 (78.8%) | | 106 (68.4%) | | | 135 (89.4%) | |  | |  | | |  | |
| Lower | 62 (20.3%) | | 47 (30.3%) | | | 15 (9.9%) | | -1.4 | | 0.3 [0.1,0.5] | | | <0.001 | |
| Higher | 3 (1.0%) | | 2 (1.3%) | | | 1 (0.7%) | | -0.9 | | 0.4 [0.0,4.2] | | | 0.45 | |
| Hematocrit, % | 38.8 [35.1,42.1] | | 36.6 [33.0,40.1] | | | 40.8 [37.9,43.2] | |  | |  | | |  | |
| Normal | 155 (50.7%) | | 41 (26.5%) | | | 114 (75.5%) | |  | |  | | |  | |
| Lower | 147 (48.0%) | | 113 (72.9%) | | | 34 (22.5%) | | -2.2 | | 0.1 [0.1,0.2] | | | <0.001 | |
| Higher | 4 (1.3%) | | 1 (0.6%) | | | 3 (2.0%) | | 0.1 | | 1.1 [0.1,22.1] | | | 0.95 | |
| Mean corpuscular volume, fL | 89.4 [86.4,92.4] | | 88.7 [85.8,91.6] | | | 90.1 [87.4,93.0] | |  | |  | | |  | |
| Normal | 283 (92.5%) | | 144 (92.9%) | | | 139 (92.1%) | |  | |  | | |  | |
| Lower | 17 (5.6%) | | 7 (4.5%) | | | 10 (6.6%) | | 0.4 | | 1.5 [0.6,4.2] | | | 0.44 | |
| Higher | 6 (2.0%) | | 4 (2.6%) | | | 2 (1.3%) | | -0.7 | | 0.5 [0.1,2.7] | | | 0.45 | |
| Mean corpuscular hemoglobin, pg | 30.7 [29.1,31.8] | | 29.7 [28.1,31.0] | | | 31.3 [30.4,32.3] | |  | |  | | |  | |
| Normal | 256 (83.7%) | | 115 (74.2%) | | | 141 (93.4%) | |  | |  | | |  | |
| Lower | 27 (8.8%) | | 20 (12.9%) | | | 7 (4.6%) | | -1.3 | | 0.3 [0.1,0.7] | | | 0.0061 | |
| Higher | 23 (7.5%) | | 20 (12.9%) | | | 3 (2.0%) | | -2.1 | | 0.1 [0.0,0.4] | | | 0.00088 | |
| Mean corpuscular hemoglobin concentration, g/L | 341.0 [328.0,351.0] | | 331.0 [322.0,343.0] | | | 347.0 [339.0,354.0] | |  | |  | | |  | |
| Normal | 231 (75.5%) | | 119 (76.8%) | | | 112 (74.2%) | |  | |  | | |  | |
|  |  | |  | | |  | |  | |  | | |  | |
|  |  | |  | | |  | |  | |  | | |  | |
| (Continued from previous page) | | | | | | | | | | | | | | |
|  | **All patients**  **(n=306)** | | **Influenza A**  **(n=155)** | | | **COVID-19**  **(n=151)** | | **Coef.** | | **OR [95%CI]** | | | **P** | |
| Lower | 34 (11.1%) | | 30 (19.4%) | | | 4 (2.6%) | | -2.0 | | 0.1 [0.0,0.4] | | | <0.001 | |
| Higher | 41 (13.4%) | | 6 (3.9%) | | | 35 (23.2%) | | 1.8 | | 6.2 [2.7,16.9] | | | <0.001 | |
| Coefficient variation of red blood cell volume distribution width, % | 12.8 [12.4,13.5] | | 13.2 [12.6,13.9] | | | 12.6 [12.3,13.0] | |  | |  | | |  | |
| Normal | 292 (95.4%) | | 144 (92.9%) | | | 148 (98.0%) | |  | |  | | |  | |
| Lower | 5 (1.6%) | | 3 (1.9%) | | | 2 (1.3%) | | -0.4 | | 0.6 [0.0.1,4] | | | 0.64 | |
| Higher | 9 (2.9%) | | 8 (5.2%) | | | 1 (0.7%) | | -2.1 | | 0.1 [0.0,0.7] | | | 0.048 | |
| Platelet count, × 10⁹ per L | 180.0 [145.0,234.0] | | 185.0 [156.0,235.0] | | | 177.0 [140.0,233.0] | |  | |  | | |  | |
| Normal | 261 (85.3%) | | 138 (89.0%) | | | 123 (81.5%) | |  | |  | | |  | |
| Lower | 29 (9.5%) | | 5 (3.2%) | | | 24 (15.9%) | | 1.7 | | 5.4 [2.2,16.4] | | | 0.00090 | |
| Higher | 16 (5.2%) | | 12 (7.7%) | | | 4 (2.6%) | | -1.0 | | 0.4 [0.1,1.1] | | | 0.096 | |
| Mean platelet volume, fL | 10.8 [10.2,11.5] | | 10.6 [9.80,11.4] | | | 11.0 [10.4,11.6] | |  | |  | | |  | |
| Normal | 290 (94.8%) | | 147 (94.8%) | | | 143 (94.7%) | |  | |  | | |  | |
| Lower | 2 (0.7%) | | 2 (1.3%) | | | 0 (0%) | | -14.5 | | 0.0 [NA,1.74805440991176E+36] | | | 0.98 | |
| Higher | 14 (4.6%) | | 6 (3.9%) | | | 8 (5.3%) | | 0.3 | | 1.4 [0.5,4.3] | | | 0.57 | |
| Platelet distribution width, % | 12.8 [11.5,14.4] | | 12.6 [11.4,14.5] | | | 12.9 [11.9,14.3] | |  | |  | | |  | |
| Normal | 285 (93.1%) | | 143 (92.3%) | | | 142 (94.0%) | |  | |  | | |  | |
|  |  | |  | | |  | |  | |  | | |  | |
|  |  | |  | | |  | |  | |  | | |  | |
| (Continued from previous page) | | | | | | | | | | | | | | |
|  | **All patients**  **(n=306)** | | **Influenza A**  **(n=155)** | | | **COVID-19**  **(n=151)** | | **Coef.** | | **OR [95%CI]** | | | **P** | |
| Lower | 3 (1.0%) | | 3 (1.9%) | | | 0 (0%) | | -15.6 | | 0.0 [NA,7.0780689004466E+40] | | | 0.99 | |
| Higher | 18 (5.9%) | | 9 (5.8%) | | | 9 (6.0%) | | 0.0 | | 1.0 [0.4,2.7] | | | 0.99 | |
| Thrombocytocrit, % | 0.2 [0.2,0.2] | | 0.2 [0.2,0.2] | | | 0.2 [0.2,0.3] | |  | |  | | |  | |
| Normal | 246 (80.4%) | | 125 (80.6%) | | | 121 (80.1%) | |  | |  | | |  | |
| Lower | 17 (5.6%) | | 10 (6.5%) | | | 7 (4.6%) | | -0.3 | | 0.7 [0.3,1.9] | | | 0.52 | |
| Higher | 43 (14.1%) | | 20 (12.9%) | | | 23 (15.2%) | | 0.2 | | 1.2 [0.6,2.3] | | | 0.60 | |
| Total protein, g/L | 68.3 [62.8,72.9] | | 63.2 [59.3,66.9] | | | 72.3 [68.9,75.7] | |  | |  | | |  | |
| Normal | 240/303 (79.2%) | | 106/154 (68.8%) | | | 134/149 (89.9%) | |  | |  | | |  | |
| Lower | 58/303 (19.1%) | | 47/154 (30.5%) | | | 11/149 (7.4%) | | -1.7 | | 0.2 [0.1,0.4] | | | <0.001 | |
| Higher | 5/303 (1.67%) | | 1/154 (0.6%) | | | 4/149 (2.7%) | | 1.2 | | 3.2 [0.5,62.4] | | | 0.30 | |
| Albumin, g/L | 38.9 [36.0,41.7] | | 37.3 [33.7,39.7] | | | 40.9 [38.1,43.6] | |  | |  | | |  | |
| Normal | 169/305 (55.4%) | | 79/154 (51.3%) | | | 90 (59.6%) | |  | |  | | |  | |
| Lower | 136/305 (44.6%) | | 75/154 (48.7%) | | | 61 (40.4%) | | -0.3 | | 0.7 [0.5,1.1] | | | 0.15 | |
| Globulin, g/L | 28.9 [25.3,32.7] | | 25.7 [23.3,29.1] | | | 31.6 [28.8,34.4] | |  | |  | | |  | |
| Normal | 289/305 (94.8%) | | 146/154 (94.8%) | | | 143 (94.7%) | |  | |  | | |  | |
| Lower | 5/305 (1.6%) | | 5/154 (3.2%) | | | 0 (0%) | | -15.5 | | 0.0 [NA,4.01024294989862E+22] | | | 0.98 | |
| Higher | 11/305 (3.6%) | | 3/154 (1.9%) | | | 8 (5.3%) | | 1.0 | | 2.7 [0.8,12.6] | | | 0.15 | |
|  | | | | | | | | | | | | | | |
|  | | | | | | | | | | | | | | |
| (Continued from previous page) | | | | | | | | | | | | | | |
|  | **All patients**  **(n=306)** | | **Influenza A**  **(n=155)** | | | **COVID-19**  **(n=151)** | | **Coef.** | | **OR [95%CI]** | | | **P** | |
| Albumin/Globulin | 1.4 [1.2,1.5] | | 1.4 [1.2,1.6] | | | 1.3 [1.2,1.5] | |  | |  | | |  | |
| Normal | 229 (74.8%) | | 113 (72.9%) | | | 116 (76.8%) | |  | |  | | |  | |
| Lower | 76 (24.8%) | | 41 (26.5%) | | | 35 (23.2%) | | -0.2 | | 0.8 [0.5,1.4] | | | 0.49 | |
| Prealbumin, g/L | 18.6 [14.0,24.2] | | 17.2 [13.6,21.1] | | | 20.8 [14.7,28.5] | |  | |  | | |  | |
| Normal | 131/301 (43.5%) | | 44/150 (29.3%) | | | 87 (57.6%) | |  | |  | | |  | |
| Lower | 167/301 (55.5%) | | 104/150 (69.3%) | | | 63 (41.7%) | | -1.2 | | 0.3 [0.2,0.5] | | | <0.001 | |
| Higher | 3/301 (1.0%) | | 2/150 (1.3%) | | | 1 (0.7%) | | -1.4 | | 0.3 [0,2.7] | | | 0.27 | |
| Alanine aminotransferase, U/L | 20.0 [13.0,33.0] | | 17.0 [12.0,23.8] | | | 25.0 [15.5,37.5] | |  | |  | | |  | |
| Normal | 268/305 (87.9%) | | 143/154 (92.9%) | | | 125 (82.8%) | |  | |  | | |  | |
| Lower | 2/305 (0.7%) | | 0/154 (0%) | | | 2 (1.3%) | | 14.7 | | 2423197.9 [0.0, NA] | | | 0.98 | |
| Higher | 35/305 (11.5%) | | 11/154 (7.1%) | | | 24 (15.9%) | | 0.9 | | 2.5 [1.2,5.5] | | | 0.017 | |
| Aspartate aminotransferase, U/L | 24.0 [19.0,32.8] | | 22.0 [17.0,29.0] | | | 26.0 [20.0,33.5] | |  | |  | | |  | |
| Normal | 256 (83.7%) | | 141 (91.0%) | | | 115 (76.2%) | |  | |  | | |  | |
| Lower | 6 (2.0%) | | 0 (0%) | | | 6 (4.0%) | | 15.8 | | 7059579.2 [0, NA] | | | 0.98 | |
| Higher | 44 (14.4%) | | 14 (9.0%) | | | 30 (19.9%) | | 1.0 | | 2.6 [1.4,5.3] | | | 0.0054 | |
| AST/ALT | 1.2 [0.9,1.6] | | 1.3 [1.0,1.7] | | | 1.1 [0.8,1.4] | |  | |  | | |  | |
| Normal | 161/305 (52.8%) | | 82/154 (53.2%) | | | 79 (52.3%) | |  | |  | | |  | |
| Lower | 58/305 (19.0%) | | 17/154 (11.0%) | | | 41 (27.2%) | | 0.9 | | 2.5 [1.3,4.9] | | | 0.0053 | |
| Higher | 86/305 (28.2%) | | 55/154 (35.7%) | | | 31 (20.5%) | | -0.5 | | 0.6 [0.3,1.0] | | | 0.051 | |
| γ-glutamyl transpeptidase, U/L | 21.0 [13.0,38.0] | | 17.5 [11.0,29.0] | | | 26.0 [16.5,44.0] | |  | |  | | |  | |
| Normal | 251/305 (82.3%) | | 135/154 (87.7%) | | | 116 (76.8%) | |  | |  | | |  | |
|  |  | |  | | |  | |  | |  | | |  | |
|  |  | |  | | |  | |  | |  | | |  | |
| (Continued from previous page) | | | | | | | | | | | | | | |
|  | **All patients**  **(n=306)** | | **Influenza A**  **(n=155)** | | | **COVID-19**  **(n=151)** | | **Coef.** | | **OR [95%CI]** | | | **P** | |
| Lower | 3/305 (1.0%) | | 0/154 (0%) | | | 3 (2.0%) | | 15.7 | | 6700902.9 [0.0, NA] | | | 0.99 | |
| Higher | 51/305 (16.7%) | | 19/154 (12.3%) | | | 32 (21.2%) | | 0.7 | | 2.0 [1.1,3.7] | | | 0.033 | |
| Alkaline phosphatase, U/L | 64.0 [51.0,81.0] | | 74.0 [53.3,98.0] | | | 62.0 [49.5,69.5] | |  | |  | | |  | |
| Normal | 279/305 (91.5%) | | 137/154 (89.0%) | | | 142 (94.0%) | |  | |  | | |  | |
| Lower | 13/305 (4.3%) | | 5/154 (3.2%) | | | 8 (5.3%) | | 0.4 | | 1.5 [0.5,5.2] | | | 0.46 | |
| Higher | 13/305 (4.3%) | | 12/154 (7.8%) | | | 1 (0.7%) | | -2.5 | | 0.1 [0,0.4] | | | 0.016 | |
| Total biliary acid, *μ*mol/L | 5.30 [4.00,8.00] | | 5.70 [4.00,8.00] | | | 5.00 [3.30,7.90] | |  | |  | | |  | |
| Normal | 236/286 (82.5%) | | 121/147 (82.3%) | | | 115/139 (82.7%) | |  | |  | | |  | |
| Higher | 50/286 (17.5%) | | 26/147 (17.7%) | | | 24/139 (17.3%) | | 0.0 | | 1.0 [0.5,1.8] | | | 0.93 | |
| Total bilirubin, *μ*mol/L | 9.20 [6.70,13.4] | | 8.60 [6.10,11.7] | | | 10.9 [7.15,15.6] | |  | |  | | |  | |
| Normal | 289/304 (95.1%) | | 148/153 (96.7%) | | | 141 (93.4%) | |  | |  | | |  | |
| Higher | 15/304 (4.9%) | | 5/153 (3.3%) | | | 10 (6.6%) | | 0.7 | | 2.1 [0.7,6.9] | | | 0.19 | |
| Direct bilirubin, *μ*mol/L | 3.70 [2.71,5.00] | | 3.30 [2.43,4.30] | | | 4.10 [3.05,5.35] | |  | |  | | |  | |
| Normal | 289/305 (94.8%) | | 148/154 (96.1%) | | | 141 (93.4%) | |  | |  | | |  | |
| Higher | 16/305 (5.2%) | | 6/154 (3.9%) | | | 10 (6.6%) | | 0.6 | | 1.7 [0.6,5.3] | | | 0.29 | |
| Indirect bilirubin, *μ*mol/L | 5.50 [3.70,8.70] | | 5.35 [3.43,7.55] | | | 6.20 [3.90,10.4] | |  | |  | | |  | |
| Normal | 272/305 (89.2%) | | 147/154 (95.5%) | | | 125 (82.8%) | |  | |  | | |  | |
| Higher | 33/305 (10.8%) | | 7/154 (4.5%) | | | 26 (17.2%) | | 1.5 | | 4.4 [1.9,11.2] | | | 0.00087 | |
| Urea nitrogen, mmol/L | 3.90 [3.00,5.30] | | 4.20 [2.80,6.10] | | | 3.80 [3.05,4.70] | |  | |  | | |  | |
| Normal | 253/305 (83.0%) | | 132/154 (85.7%) | | | 121 (80.1%) | |  | |  | | |  | |
| Lower | 36/305 (11.8%) | | 9/154 (5.8%) | | | 27 (17.9%) | | 1.2 | | 3.3 [1.5,7.6] | | | 0.0034 | |
|  |  | |  | | |  | |  | |  | | |  | |
|  |  | |  | | |  | |  | |  | | |  | |
| (Continued from previous page) | | | | | | | | | | | | | | |
|  | **All patients**  **(n=306)** | | **Influenza A**  **(n=155)** | | | **COVID-19**  **(n=151)** | | **Coef.** | | **OR [95%CI]** | | | **P** | |
| Higher | 16/305 (5.2%) | | 13/154 (8.4%) | | | 3 (2.0%) | | -1.4 | | 0.3 [0.1,0.8] | | | 0.035 | |
| Creatinine, *μ*mol/L | 61.0 [50.0,77.0] | | 59.0 [46.0,76.0] | | | 65.0 [53.0,77.0] | |  | |  | | |  | |
| Normal | 295/305 (96.7%) | | 146/154 (94.8%) | | | 149 (98.7%) | |  | |  | | |  | |
| Lower | 5/305 (1.6%) | | 3/154 (1.9%) | | | 2 (1.3%) | | -0.4 | | 0.7 [0.1,4.0] | | | 0.64 | |
| Higher | 5/305 (1.6%) | | 5/154 (3.2%) | | | 0 (0%) | | -15.6 | | 0.0 [NA,3.85109651699767E+22] | | | 0.98 | |
| Cystatin C, mg/L | 0.9 [0.8,1.1] | | 1.1 [0.9,1.3] | | | 0.8 [0.7,0.9] | |  | |  | | |  | |
| Normal | 243/305 (79.7%) | | 105/154 (68.2%) | | | 138 (91.4%) | |  | |  | | |  | |
| Lower | 11/305 (3.6%) | | 2/154 (1.3%) | | | 9 (6.0%) | | 1.2 | | 3.4 [0.9,22.8] | | | 0.12 | |
| Higher | 51/305 (16.7%) | | 47/154 (30.5%) | | | 4 (2.6%) | | -2.7 | | 0.1 [0.0,0.2] | | | <0.001 | |
| Uric acid, *μ*mol/L | 241.0 [197.0,301.0] | | 262.0 [220.0,315.0] | | | 230.0 [182.0,292.0] | |  | |  | | |  | |
| Normal | 243/305 (79.7%) | | 139/154 (90.3%) | | | 104 (68.9%) | |  | |  | | |  | |
| Lower | 49/305 (16.1%) | | 8/154 (5.2%) | | | 41 (27.2%) | | 1.9 | | 6.9 [3.2,16.3] | | | <0.001 | |
| Higher | 13/305 (4.3%) | | 7/154 (4.5%) | | | 6 (4.0%) | | 0.1 | | 1.1 [0.4,3.5] | | | 0.81 | |
| Retinol-Binding Protein, mg/L | 24.2 [16.8,30.2] | | 21.5 [16.0,29.4] | | | 25.1 [19.0,31.0] | |  | |  | | |  | |
| Normal | 131 (42.8%) | | 58 (37.4%) | | | 73 (48.3%) | |  | |  | | |  | |
| Lower | 149 (48.7%) | | 83 (53.5%) | | | 66 (43.7%) | | -0.5 | | 0.6 [0.4,1] | | | 0.057 | |
| Higher | 2 (0.7%) | | 2 (1.3%) | | | 0 (0%) | | -14.8 | | 0.0 [NA,1.32264605120244E+36] | | | 0.98 | |
|  |  | |  | | |  | |  | |  | | |  | |
|  |  | |  | | |  | |  | |  | | |  | |
| (Continued from previous page) | | | | | | | | | | | | | | |
|  | **All patients**  **(n=306)** | | **Influenza A**  **(n=155)** | | | **COVID-19**  **(n=151)** | | **Coef.** | | **OR [95%CI]** | | | **P** | |
| Glucose, mmol/L | 5.8 [5.1,6.7] | | 5.4 [4.8,6.1] | | | 6.2 [5.6,7.0] | |  | |  | | |  | |
| Normal | 184/305 (60.3%) | | 112/154 (72.7%) | | | 72 (47.7%) | |  | |  | | |  | |
| Lower | 4/305 (1.3%) | | 4/154 (2.6%) | | | 0 (0%) | | -15.1 | | 0.0 [NA,4.50801336129706E+29] | | | 0.98 | |
| Higher | 117/305 (38.4%) | | 38/154 (24.7%) | | | 79 (52.3%) | | 1.2 | | 3.2 [2,5.3] | | | <0.001 | |
| Total cholesterol, mmol/L | 3.8 [3.3,4.6] | | 3.9 [3.3,4.7] | | | 3.7 [3.4,4.3] | |  | |  | | |  | |
| Normal | 238/286 (83.2%) | | 112/147 (76.2%) | | | 126/139 (90.6%) | |  | |  | | |  | |
| Lower | 16/286 (5.6%) | | 11/147 (7.5%) | | | 5/139 (3.6%) | | -0.9 | | 0.4 [0.1,1.1] | | | 0.10 | |
| Higher | 32/286 (11.2%) | | 24/147 (16.3%) | | | 8/139 (5.8%) | | -1.2 | | 0.3 [0.1,0.7] | | | 0.0045 | |
| Triglyceride, mmol/L | 1.1 [0.8,1.6] | | 1.0 [0.7,1.5] | | | 1.2 [0.9,1.7] | |  | |  | | |  | |
| Normal | 211/286 (73.8%) | | 111/147 (75.5%) | | | 100/139 (71.9%) | |  | |  | | |  | |
| Lower | 27/286 (9.4%) | | 21/147 (14.3%) | | | 6/139 (4.3%) | | -1.1 | | 0.3 [0.1,0.8] | | | 0.017 | |
| Higher | 48/286 (16.8%) | | 15/147 (10.2%) | | | 33/139 (23.7%) | | 0.9 | | 2.4 [1.3,4.9] | | | 0.0088 | |
| High-density lipoprotein cholesterol, mmol/L | 1.1 [0.9,1.4] | | 1.3 [1.0,1.4] | | | 1.0 [0.8,1.1] | |  | |  | | |  | |
| Normal | 136/285 (44.7%) | | 85/147 (57.8%) | | | 51/130 (39.2%) | |  | |  | | |  | |
| Lower | 125/285 (43.9%) | | 45/147 (30.6%) | | | 80/130 (61.5%) | | 1.1 | | 3.0 [1.8,4.9] | | | <0.001 | |
| Higher | 24/285 (8.4%) | | 17/147 (11.6%) | | | 7/130 (5.4%) | | -0.4 | | 0.7 [0.3,1.7] | | | 0.44 | |
| Low-density lipoprotein cholesterol, mmol/L | 2.1 [1.7,2.7] | | 2.0 [1.6,2.7] | | | 2.1 [1.8,2.7] | |  | |  | | |  | |
|  |  | |  | | |  | |  | |  | | |  | |
|  |  | |  | | |  | |  | |  | | |  | |
| (Continued from previous page) | | | | | | | | | | | | | | |
|  | **All patients**  **(n=306)** | | **Influenza A**  **(n=155)** | | | **COVID-19**  **(n=151)** | | **Coef.** | | **OR [95%CI]** | | | **P** | |
| Normal | 174/285 (61.1%) | | 47/147 (32.0%) | | | 127/130 (97.7%) | |  | |  | | |  | |
| Lower | 80/285 (28.1%) | | 80/147 (54.4%) | | | 0/130 (0.0%) | | -19.6 | | 0.0 [0,395.3] | | | 0.98 | |
| Higher | 31/285 (10.9%) | | 20/147 (13.6%) | | | 11/130 (8.5%) | | -1.6 | | 0.2 [0.1,0.4] | | | <0.001 | |
| Apolipoprotein A1, g/L | 1.1 [1.0,1.3] | | 1.2 [1.0,1.5] | | | 1.1 [1.0,1.2] | |  | |  | | |  | |
| Normal | 160/285 (56.1%) | | 78/147 (53.1%) | | | 82/130 (63.1%) | |  | |  | | |  | |
| Lower | 96/285 (33.7%) | | 43/147 (29.3%) | | | 53/130 (40.8%) | | 0.2 | | 1.2 [0.7,2] | | | 0.54 | |
| Higher | 29/285 (10.2%) | | 26/147 (17.7%) | | | 3/130 (2.3%) | | -2.2 | | 0.1 [0,0.3] | | | <0.001 | |
| Apolipoprotein B, g/L | 0.7 [0.6,0.9] | | 0.7 [0.6,0.9] | | | 0.8 [0.7,0.9] | |  | |  | | |  | |
| Normal | 182/267 (68.2%) | | 90/147 (61.2%) | | | 92/120 (76.7%) | |  | |  | | |  | |
| Lower | 64/267 (24.0%) | | 44/147 (29.9%) | | | 20/120 (16.7%) | | -0.8 | | 0.4 [0.2,0.8] | | | 0.0085 | |
| Higher | 21/267 (7.9%) | | 13/147 (8.8%) | | | 8/120 (6.7%) | | -0.5 | | 0.6 [0.2,1.5] | | | 0.28 | |
| Lipoprotein a, mg/L | 135.0 [54.2,257.0] | | 111.0 [28.7,246.0] | | | 139.0 [81.0,276.0] | |  | |  | | |  | |
| Normal | 205/268 (76.5.0%) | | 113/147 (76.9%) | | | 92 (76.0%) | |  | |  | | |  | |
| Higher | 63/268 (23.5%) | | 34/147 (23.1%) | | | 29 (24.0%) | | 0.0 | | 1.0 [0.6,1.8] | | | 0.87 | |
| Creatine kinase, U/L | 67.0 [45.5,110.0] | | 74.5 [45.8,136.0] | | | 61.0 [45.5,88.0] | |  | |  | | |  | |
| Normal | 212/279 (76.0%) | | 128/152 (84.2%) | | | 84/127 (66.1%) | |  | |  | | |  | |
| Lower | 42/279 (15.1%) | | 3/152 (2.0%) | | | 39/127 (30.7%) | | 3.0 | | 19.8 [6.9,83.8] | | | <0.001 | |
| Higher | 25/279 (9.0%) | | 21/152 (13.8%) | | | 4/127 (3.1%) | | -1.2 | | 0.3 [0.1,0.8] | | | 0.028 | |
| Creatine kinase-MB, U/L | 11.0 [8.00,15.0] | | 13.0 [11.0,18.0] | | | 9.00 [5.00,12.0] | |  | |  | | |  | |
| Normal | 271/297 (91.2%) | | 135/152 (88.8%) | | | 136/145 (93.8%) | |  | |  | | |  | |
| Higher | 26/297 (8.8%) | | 17/152 (11.2%) | | | 9/145 (6.2%) | | -0.6 | | 0.5 [0.2,1.2] | | | 0.13 | |
|  |  | |  | | |  | |  | |  | | |  | |
|  |  | |  | | |  | |  | |  | | |  | |
| (Continued from previous page) | | | | | | | | | | | | | | |
|  | **All patients**  **(n=306)** | | **Influenza A**  **(n=155)** | | | **COVID-19**  **(n=151)** | | **Coef.** | | **OR [95%CI]** | | | **P** | |
| Lactate dehydrogenase, U/L | 214.0 [181.0,267.0] | | 196.0 [169.0,243.0] | | | 236.0 [202.0,291.0] | |  | |  | | |  | |
| Normal | 182/298 (61.1%) | | 100/152 (65.8%) | | | 82/146 (56.2%) | |  | |  | | |  | |
| Lower | 6/298 (2.0%) | | 6/152 (3.9%) | | | 0/146 (0%) | | -15.4 | | 0.0 [NA,1144791795912550000] | | | 0.98 | |
| Higher | 110/298 (36.9%) | | 46/152 (30.3%) | | | 64/146 (43.8%) | | 0.5 | | 1.7 [1.1,2.7] | | | 0.030 | |
| α-hydroxybutyric dehydrogenase, U/L | 165.0 [142.0,201.0] | | 151.0 [128.0,185.0] | | | 177.0 [150.0,216.0] | |  | |  | | |  | |
| Normal | 189/298 (63.4%) | | 111/152 (73.0%) | | | 78/146 (53.4%) | |  | |  | | |  | |
| Higher | 109/298 (36.6%) | | 41/152 (27.0%) | | | 68/146 (46.6%) | | 0.9 | | 2.4 [1.5,3.9] | | | 0.00050 | |
| Homocysteine, *μ*mol/L | 9.9 [7.0,13.4] | | 7.8 [5.8,10.7] | | | 11.6 [9.4,14.6] | |  | |  | | |  | |
| Normal | 216/290 (74.5%) | | 113/147 (76.9%) | | | 103/143 (72.0%) | |  | |  | | |  | |
| Lower | 19/290 (6.6%) | | 17/147 (11.6%) | | | 2/143 (1.4%) | | -2.0 | | 0.1 [0.0,0.5] | | | 0.0071 | |
| Higher | 55/290 (19.0%) | | 17/147 (11.6%) | | | 38/143 (26.6%) | | 0.9 | | 2.5 [1.3,4.7] | | | 0.0053 | |
| Kalium, mmol/L | 3.8 [3.5,4.1] | | 3.9 [3.5,4.2] | | | 3.7 [3.5,4.1] | |  | |  | | |  | |
| Normal | 229/302 (75.8%) | | 118/152 (77.6%) | | | 111/150 (74.0%) | |  | |  | | |  | |
| Lower | 71/302 (23.5%) | | 34/152 (22.4%) | | | 37/150 (24.7%) | | 0.1 | | 1.2 [0.7,2.0] | | | 0.59 | |
| Higher | 2/302 (0.7%) | | 0/152 (0%) | | | 2/150 (1.3%) | | 14.6 | | 2251758.9 [0.0, NA] | | | 0.98 | |
| Natrium, mmol/L | 138.0 [136.0,140.0] | | 138.0 [136.0,140.0] | | | 137.0 [135.0,139.0] | |  | |  | | |  | |
| Normal | 197/303 (65.0%) | | 114/154 (75.0%) | | | 83 (55.0%) | |  | |  | | |  | |
| Lower | 105/303 (34.7%) | | 38/154 (25.0%) | | | 67 (44.4%) | | 0.9 | | 2.4 [1.5,4.0] | | | <0.001 | |
|  |  | |  | | |  | |  | |  | | |  | |
| (Continued from previous page) | | | | | | | | | | | | | | |
|  | **All patients**  **(n=306)** | | **Influenza A**  **(n=155)** | | | **COVID-19**  **(n=151)** | | **Coef.** | | **OR [95%CI]** | | | **P** | |
| Higher | 1/303 (0.3%) | | 0/154 (0%) | | | 1 (0.7%) | | 14.9 | | 2909307.4 [0.0, NA] | | | 0.99 | |
| Chlorine, mmol/L | 102.0 [99.1,104.0] | | 102.0 [99.3,104.0] | | | 102.0 [98.8,104.0] | |  | |  | | |  | |
| Normal | 237/303 (78.2%) | | 128/154 (84.2%) | | | 109 (72.2%) | |  | |  | | |  | |
| Lower | 64/303 (21.1%) | | 23/154 (15.1%) | | | 41 (27.2%) | | 0.7 | | 2.1 [1.2,3.8] | | | 0.011 | |
| Higher | 2/303 (0.7%) | | 1/154 (0.7%) | | | 1 (0.7%) | | 0.2 | | 1.2 [0.0,29.9] | | | 0.91 | |
| Calcium, mmol/L | 2.2 [2.1,2.3] | | 2.2 [2.1,2.3] | | | 2.3 [2.2,2.4] | |  | |  | | |  | |
| Normal | 281/303 (92.7%) | | 136/154 (89.5%) | | | 145 (96.0%) | |  | |  | | |  | |
| Lower | 22/303 (7.3%) | | 16/154 (10.5%) | | | 6 (4.0%) | | -1.0 | | 0.4 [0.1,0.9] | | | 0.034 | |
| Phosphorus, mmol/L | 1.10 [0.9,1.3] | | 1.2 [1.1,1.5] | | | 1.0 [0.9,1.1] | |  | |  | | |  | |
| Normal | 243/303 (80.2%) | | 125/154 (82.2%) | | | 118 (78.1%) | |  | |  | | |  | |
| Lower | 39/303 (12.9%) | | 8/154 (5.3%) | | | 31 (20.5%) | | 1.4 | | 4.1 [1.9,9.9] | | | 0.00070 | |
| Higher | 21/303 (6.9%) | | 19/154 (12.5%) | | | 2 (1.3%) | | -2.2 | | 0.1 [0.0,0.4] | | | 0.0036 | |
| Magnesium, mmol/L | 0.9 [0.8,1.0] | | 0.9 [0.8,1.0] | | | 0.9 [0.8,0.9] | |  | |  | | |  | |
| Normal | 264/303 (87.1%) | | 126/154 (82.9%) | | | 138 (91.4%) | |  | |  | | |  | |
| Lower | 24/303 (7.9%) | | 20/154 (13.2%) | | | 4 (2.6%) | | -1.7 | | 0.2 [0.1,0.5] | | | 0.0025 | |
| Higher | 15/303 (5.0%) | | 6/154 (3.9%) | | | 9 (6.0%) | | 0.3 | | 1.4 [0.5,4.2] | | | 0.56 | |
| Bicarbonate, mmol/L | 26.2 [23.8,28.5] | | 25.2 [22.3,27.0] | | | 26.4 [24.5,28.7] | |  | |  | | |  | |
| Normal | 170/201 (84.6%) | | 36/51 (70.6%) | | | 134/150 (89.3%) | |  | |  | | |  | |
| Lower | 24/201 (11.9%) | | 11/51 (21.6%) | | | 13/150 (8.7%) | | -1.1 | | 0.3 [0.1,0.8] | | | 0.011 | |
| Higher | 7/201 (3.5%) | | 4/51 (7.8%) | | | 3/150 (2.0%) | | -1.6 | | 0.2 [0.0,1.0] | | | 0.042 | |
|  | | | | | | | | | | | | | | |
|  | | | | | | | | | | | | | | |
|  | | | | | | | | | | | | | | |
| (Continued from previous page) | | | | | | | | | | | | | | |
|  | **All patients**  **(n=306)** | | **Influenza A**  **(n=155)** | | | **COVID-19**  **(n=151)** | | **Coef.** | | **OR [95%CI]** | | | **P** | |
| C-reactive protein, mg/L | 24.4 [8.15,55.6] | | 38.7 [19.8,66.8] | | | 14.8 [3.20,35.6] | |  | |  | | |  | |
| Normal | 55/291 (18.9%) | | 9/146 (6.2%) | | | 46/145 (31.7%) | |  | |  | | |  | |
| Higher | 236/291 (81.1%) | | 137/146 (93.8%) | | | 99/145 (68.3%) | | -2.0 | | 0.1 [0.1,0.3] | | | <0.001 | |
| Procalcitonin ng/mL | 0.0 [0.0,0.1] | | 0.1 [0.0,0.2] | | | 0.0 [0.0,0.1] | |  | |  | | |  | |
| Normal | 157/165 (95.2%) | | 16/21 (76.2%) | | | 141/144 (97.9%) | |  | |  | | |  | |
| Higher | 8/165 (4.8%) | | 5/21 (23.8%) | | | 3/144 (2.1%) | | -2.7 | | 0.1 [0.0,0.3] | | | 0.00054 | |
| **Data are median (IQR), n (%), or n/N (%), where N is the total number of patients with available data. Comparing COVID-19 and influenza A are from logistic regression.**  **Abbreviations: Coef. = coefficient; OR = odds ratio; CI = confidence interval.** | | | | | | | | | | | | | | |
