# Supplementary material for: Triage Modeling for Differential Diagnosis Between COVID-19 and Human Influenza A Pneumonia: Classification and Regression Tree Analysis
Source: Front Med (Lausanne). 2021 Aug 10;8:673253. doi: 10.3389/fmed.2021.673253 (PMC8382719; doi:10.3389/fmed.2021.673253)
Supplement: Supplementary file 2 [file Data_Sheet_1.docx]

**Triage modeling for differential diagnosis between COVID-19 and Human Influenza A pneumonia: Classification and regression tree analysis**

Anling Xiao ^1, †^, Huijuan Zhao ^2, 3,^ ^†^, Jianbing Xia ^4,^ ^†^, Ling Zhang ^4^, Chao Zhang ^2, 3^, Zhuoying Ruan ^5^, Nan Mei ^6^, Xun Li ^2, 3^, Wuren Ma ^2, 3^, Zhuozhu Wang ^7^, Yi He ^8^, Jimmy Lee ^9^, Weiming Zhu ^10^, Dajun Tian ^11^, Kunkun Zhang^12^, Weiwei Zheng ^2, 3,^ * & Bo Yin ^6^, *

^1^ Department of radiology, Fu Yang No.2 People`s Hospital, 450 Linquan Road, Fuyang, Anhui Province 236015, China.

^2^ Key Laboratory of Public Health Safety, Ministry of Education, Department of Environmental Health, School of Public Health, Fudan University, Shanghai 200032, China.

^3^ Key Laboratory of Health Technology Assessment, National Health Commission of the People’s Republic of China, Fudan University, Shanghai 200032, China.

^4^ Shanghai Ninth People's Hospital, Shanghai JiaoTong University School of Medicine, Shanghai 200011, China.

^5^ Department of radiology, Shanghai Institute of Medical Imaging, Shanghai 200032, China.

^6^ Huashan Hospital, Fudan University, 12 Middle Wulumuqi Rd., Shanghai 200040, China.

^7^ Department of Psychology, Zhejiang University, 866 Yuhangtang Road, Hangzhou, Zhejiang 310058, China.

^8^ Curtin University of Technology, (WA) Australia, Kent St. Bentley WA 6102, Australia.

^9^ Department of Management, University of California, Los Angeles, 405 Hilgard Avenue, Los Angeles, California 90095, USA.

^10^ Department of Epidemiology, Los Angeles, University of California, 650 Charles E Young Dr S, Los Angeles, CA 90095, USA.

^11^ Department of Epidemiology and Biostatistics, College for Public Health and Social Justice, Saint Louis University, 3545 Lafayette Ave.St. Louis, MO 63104, USA.

^12^ Department of Finance, Shanghai Children’s Hospital, Shanghai Jiao Tong University,355 Luding Road, Putuo District, Shanghai 200062, China.

^†^ These authors contributed equally to these work.

^*^ Correspondence to: Tel./Fax: 086-021-54237229. Postal address: No. 130 Dongan Road, Shanghai 200032, China. E-mail addresses: [weiweizheng@fudan.edu.cn](mailto:weiweizheng@fudan.edu.cn) (Weiwei Zheng); Huashan Hospital, Fudan University, 12 Middle Wulumuqi Rd., Shanghai 200040, China. E-mail addresses: [yinbo@fudan.edu.cn](mailto:yinbo@fudan.edu.cn) (Bo Yin).

**Content**

[A. Flow chart for model derivation and validation](#_Toc26302)

[B. Model C (signs and symptoms)](#_Toc16164)

[C. Model D (routine blood)](#_Toc25184)

[D. Model E (serum biochemistry)](#_Toc29197)

[E. Model F (signs and symptoms + blood routine + serum biochemistry)](#_Toc6871)

# Flow chart for model derivation and validation

**
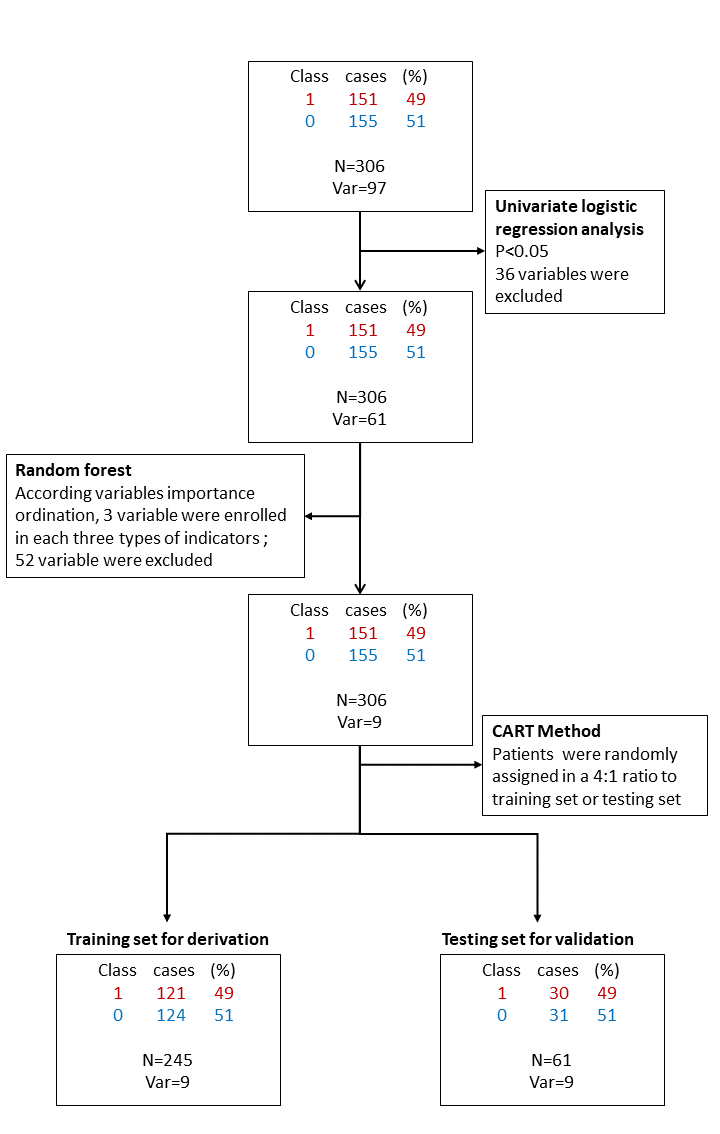
**

**Figure. S1: The main methods of model derivation and validation,** **stratified by cases in** **COVID-19 and** **Influenza A.**

0=Influenza A; 1=COVID-19; N= the total number of patients; Var=the number of variables.

# Model C (signs and symptoms)


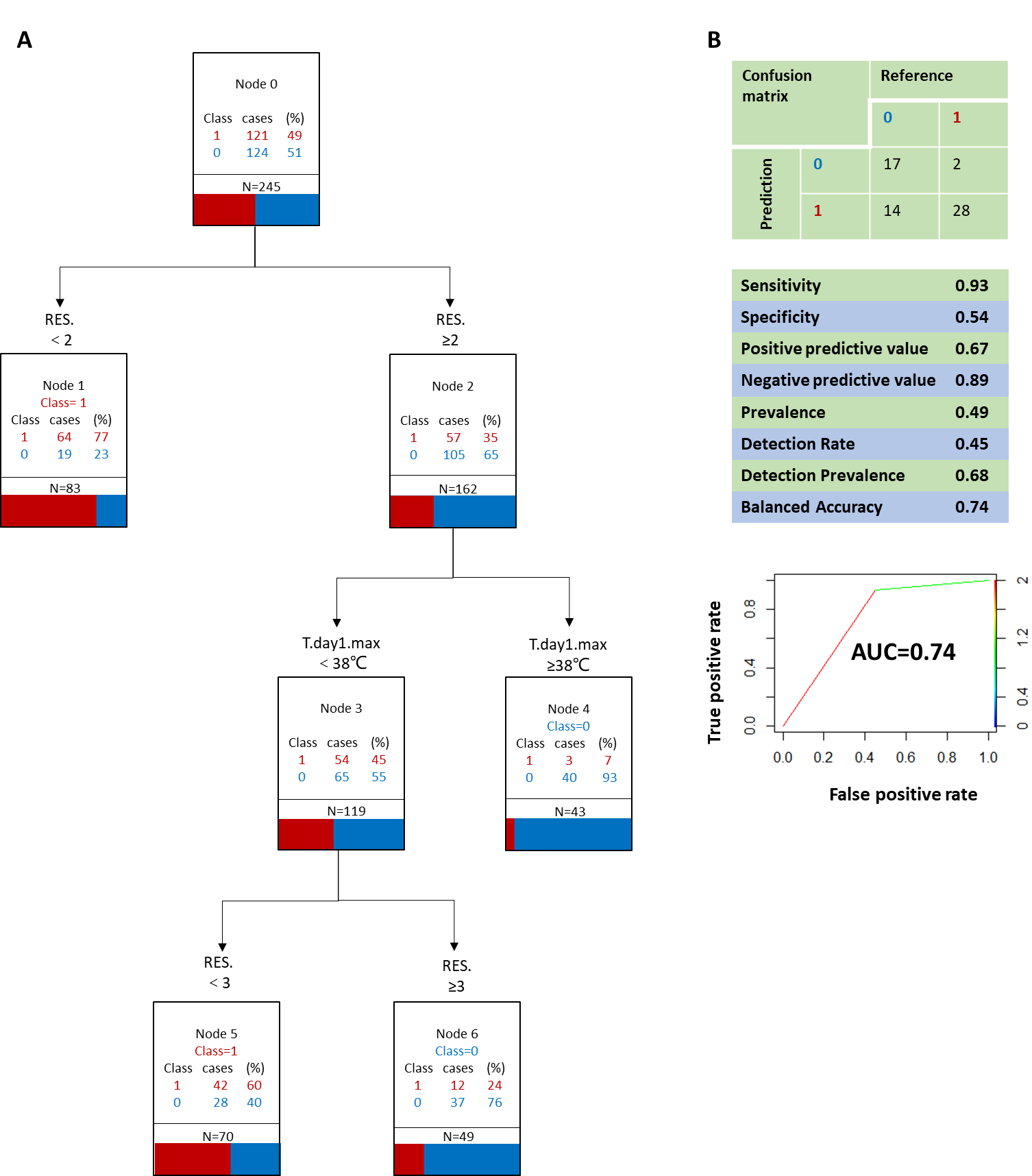


**Figure. S2: Classification and regression tree analysis of variables that most distinguish COVID-19 from Influenza A in clinical signs and symptoms.**

1. 0=Influenza A; 1=COVID-19; N= the total number of patients; RES.= the number of respiratory symptom; T.day1.max=Highest temperature on the first day of admission. All factors are compared with the limit of the range of medical reference value. (B) Performance characteristics of the model validated by the testing set.

# Model D (routine blood)


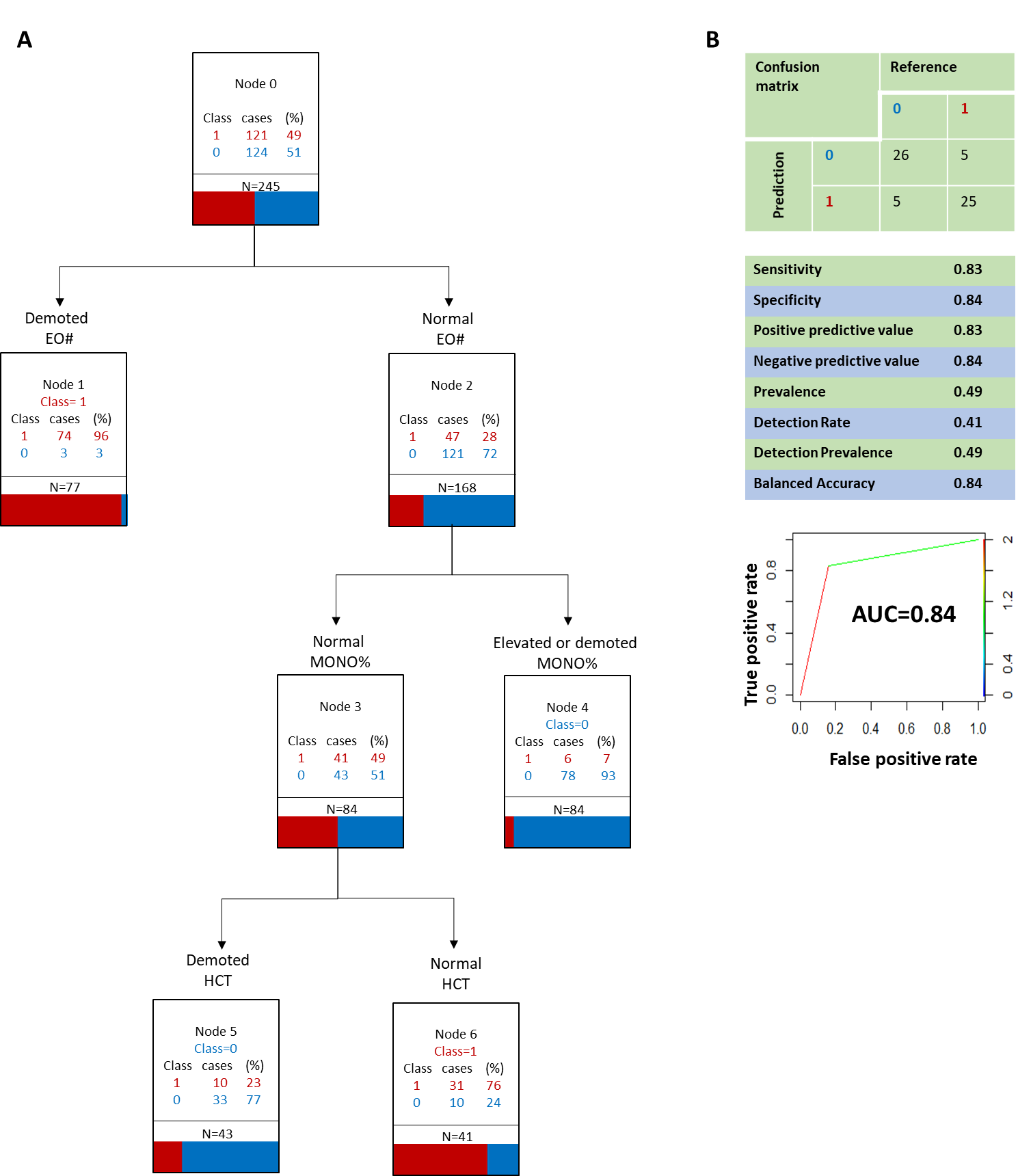


**Figure. S3: Classification and regression tree analysis of variables that most distinguish COVID-19 from Influenza A in blood routine.**

(A) 0=Influenza A; 1=COVID-19; N= the total number of patients; EO#=eosinophil count; MONO%=monocyte ratio; HCT=Hematocrit. All factors are compared with the limit of the range of medical reference value. (B) Performance characteristics of the model validated by the testing set.

# Model E (serum biochemistry)


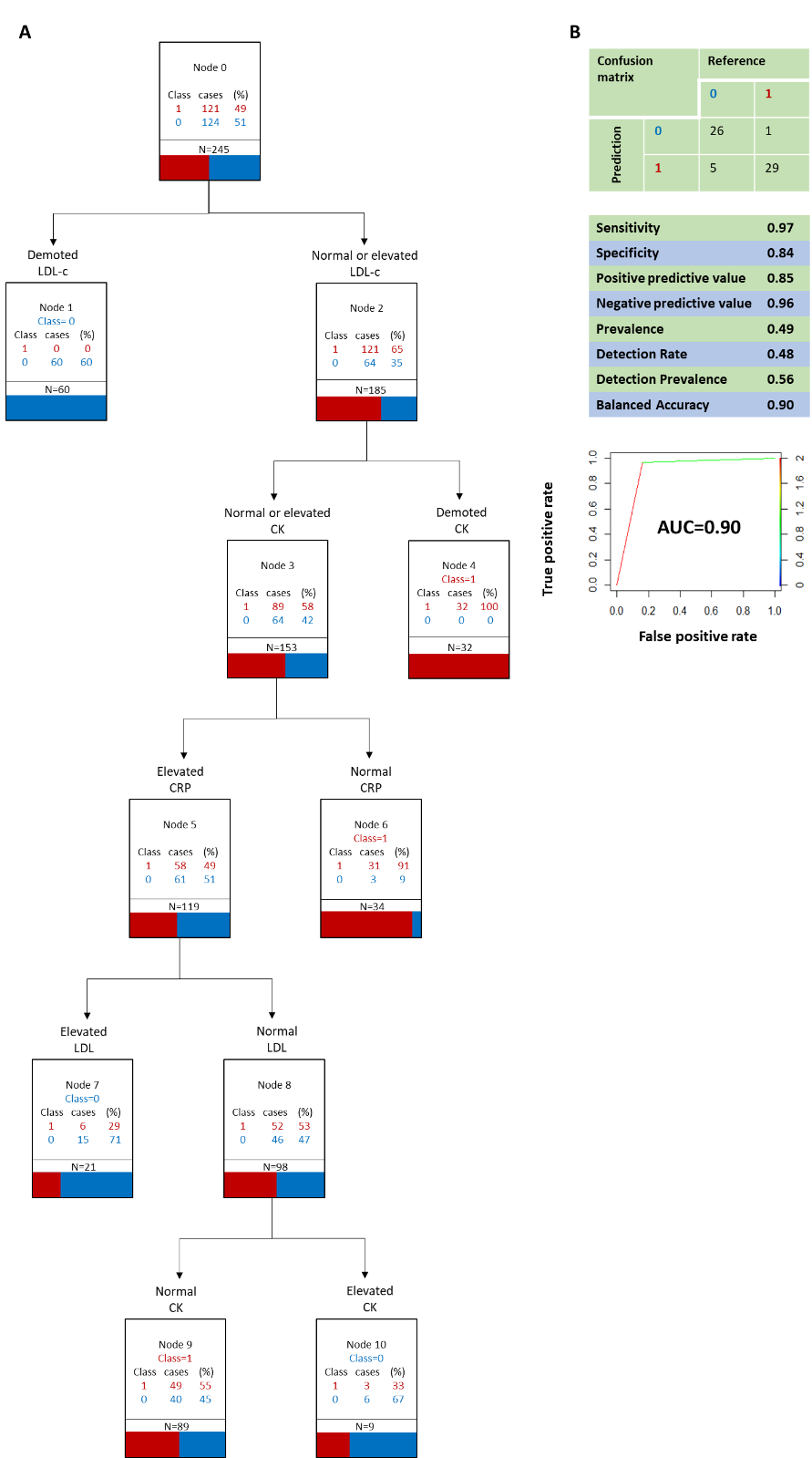


**Figure. S4: Classification and regression tree analysis of variables that most distinguish COVID-19 from Influenza A in serum biochemistry.**

1. 0=Influenza A; 1=COVID-19; N= the total number of patients; LDL-c=Low-density lipoprotein cholesterol; CK=creatine kinase; CRP=C-reactive protein. All factors are compared with the limit of the range of medical reference value. (B) Performance characteristics of the model validated by the testing set.

# Model F (symptoms and signs + blood routine + serum biochemistry)


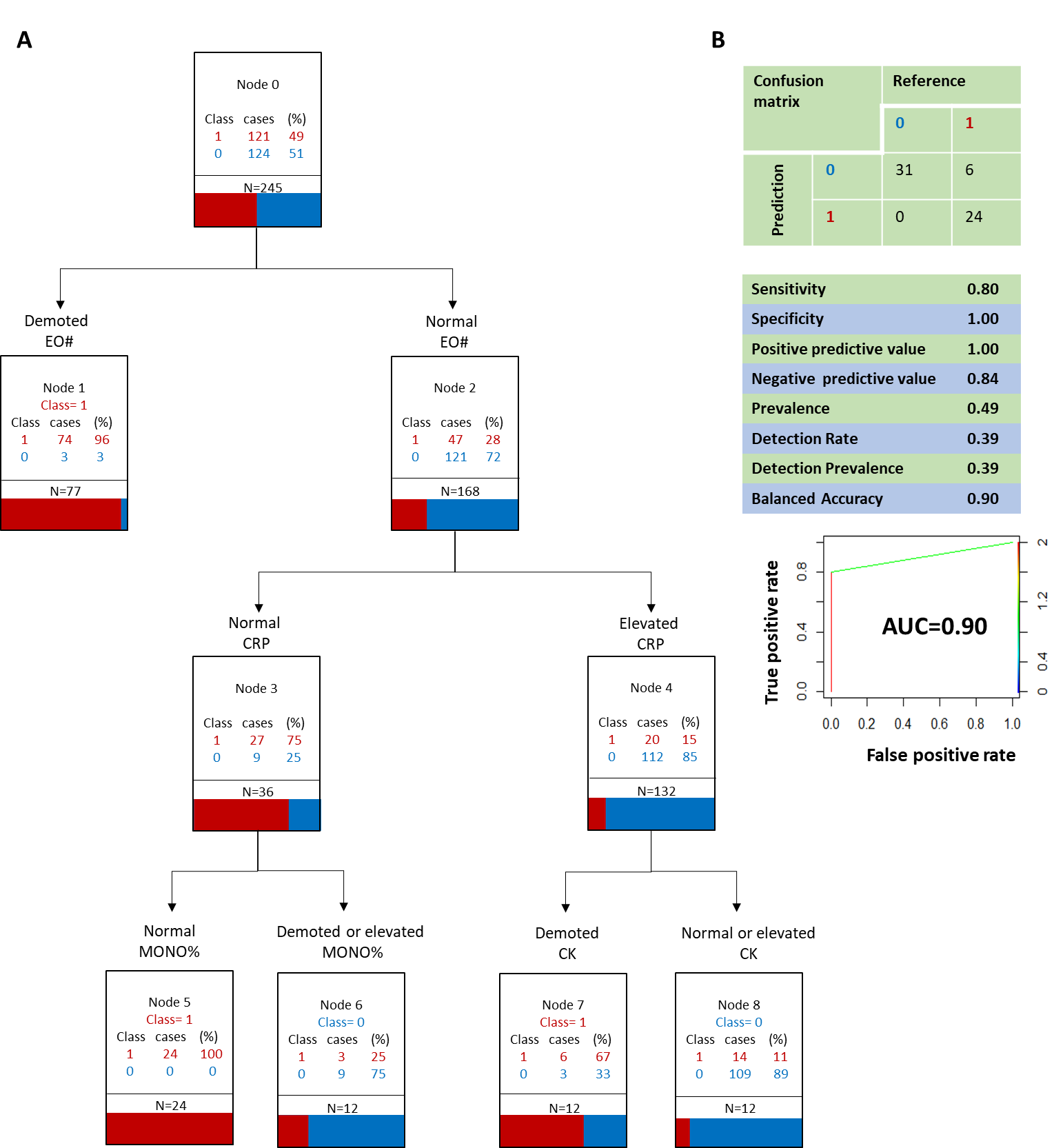


**Figure. S5: Classification and regression tree analysis of variables that most distinguish COVID-19 from Influenza A in clinical signs and symptoms, blood routine and serum biochemistry.**

(A) 0=Influenza A; 1=COVID-19; N= the total number of patients; EO#=eosinophil count; MONO%=monocyte ratio; CK=creatine kinase; CRP=C-reactive protein. All factors are compared with the limit of the range of medical reference value. (B) Performance characteristics of the model validated by the testing set.
